# A Phase 1 Trial of MSP2-C1, a Blood-Stage Malaria Vaccine Containing 2 Isoforms of MSP2 Formulated with Montanide® ISA 720

**DOI:** 10.1371/journal.pone.0024413

**Published:** 2011-09-19

**Authors:** James S. McCarthy, Joanne Marjason, Suzanne Elliott, Paul Fahey, Gilles Bang, Elissa Malkin, Eveline Tierney, Hayley Aked-Hurditch, Christopher Adda, Nadia Cross, Jack S. Richards, Freya J. I. Fowkes, Michelle J. Boyle, Carole Long, Pierre Druilhe, James G. Beeson, Robin F. Anders

**Affiliations:** 1 Queensland Institute of Medical Research (QIMR), University of Queensland, Brisbane, Australia; 2 Q-Pharm Pty Ltd, Brisbane, Australia; 3 PATH Malaria Vaccine Initiative, Bethesda, Maryland, United States of America; 4 Institut Pasteur, Paris, France; 5 Department of Biochemistry, La Trobe University, Melbourne, Australia; 6 Walter and Eliza Hall Institute, Parkville, Victoria, Australia; 7 National Institute of Allergy and Infectious Diseases, National Institutes of Health, Bethesda, Maryland, United States of America; The George Washington University Medical Center, United States of America

## Abstract

**Background:**

In a previous Phase 1/2b malaria vaccine trial testing the 3D7 isoform of the malaria vaccine candidate Merozoite surface protein 2 (MSP2), parasite densities in children were reduced by 62%. However, breakthrough parasitemias were disproportionately of the alternate dimorphic form of MSP2, the FC27 genotype. We therefore undertook a dose-escalating, double-blinded, placebo-controlled Phase 1 trial in healthy, malaria-naïve adults of MSP2-C1, a vaccine containing recombinant forms of the two families of *msp2* alleles, 3D7 and FC27 (*Ec*MSP2-3D7 and *Ec*MSP2-FC27), formulated in equal amounts with Montanide® ISA 720 as a water-in-oil emulsion.

**Methodology/Principal Findings:**

The trial was designed to include three dose cohorts (10, 40, and 80 µg), each with twelve subjects receiving the vaccine and three control subjects receiving Montanide® ISA 720 adjuvant emulsion alone, in a schedule of three doses at 12-week intervals. Due to unexpected local reactogenicity and concern regarding vaccine stability, the trial was terminated after the second immunisation of the cohort receiving the 40 µg dose; no subjects received the 80 µg dose. Immunization induced significant IgG responses to both isoforms of MSP2 in the 10 µg and 40 µg dose cohorts, with antibody levels by ELISA higher in the 40 µg cohort. Vaccine-induced antibodies recognised native protein by Western blots of parasite protein extracts and by immunofluorescence microscopy. Although the induced anti-MSP2 antibodies did not directly inhibit parasite growth *in vitro*, IgG from the majority of individuals tested caused significant antibody-dependent cellular inhibition (ADCI) of parasite growth.

**Conclusions/Significance:**

As the majority of subjects vaccinated with MSP2-C1 developed an antibody responses to both forms of MSP2, and that these antibodies mediated ADCI provide further support for MSP2 as a malaria vaccine candidate. However, in view of the reactogenicity of this formulation, further clinical development of MSP2-C1 will require formulation of MSP2 in an alternative adjuvant.

**Trial Registration:**

Australian New Zealand Clinical Trials Registry 12607000552482

## Introduction

Half of the world's population is at risk of malaria, with an estimated 225 million cases occurring in 2009, leading to an estimated 863,000 million deaths [1]. Most malaria cases occur in Africa, with 85% of an estimated 781,000 deaths in children under five years of age due to *Plasmodium falciparum* malaria [1]. Although significant advances have been made in the control of malaria through the deployment of insecticide-treated bed nets and artemisinin combination therapy (ACT) [1], the growing threat of drug resistance in malaria parasites [2] and the limited choice of insecticides necessitate the development of new tools to reduce the morbidity and mortality caused by this disease. A Phase 3 trial of the pre-erythrocytic circumsporozoite protein (CSP) vaccine RTS,S is currently ongoing, and has the potential to be the first licensed malaria vaccine. (ClinicalTrials.gov Identifier: NCT00866619). However, this vaccine is unlikely to confer complete protection. For this reason the continued clinical development of asexual blood-stage antigens as complementary or alternative vaccine components, remains a priority.

Of the life-cycle stages of the malaria parasite present in the blood that could be targeted by vaccine-induced immune responses, the merozoite has been the focus of most research. This is the life-cycle stage released when the infected erythrocyte ruptures, and is present only transiently in the blood before it invades another erythrocyte. Early experimental data demonstrating effective vaccination with whole merozoite extracts was followed by the identification of a number of important merozoite surface proteins, some of which remain vaccine candidates. These include Merozoite Surface Protein 1 (MSP1), Apical Membrane Antigen1 (AMA1), MSP2 (Merozoite Surface Protein 2) and MSP3 [3], [4], [5]. MSP2 is a ∼28 kDa protein that is anchored in the membrane of the merozoite by a C-terminal glycosylphosphatidyl-inositol (GPI) anchor [6], [7]. The function of MSP2 is unknown but it appears to be essential for parasite viability, as attempts to “knock-out” the gene have not been successful [8], [9].

Like many abundantly expressed surface proteins of the malaria parasite, MSP2 is highly polymorphic. Polymorphisms are largely confined to the central variable region of the protein, which is flanked by a short N-terminal, and a longer C-terminal conserved region [10], [11]. The variable region consists of highly polymorphic tandem sequence repeats and flanking non-repetitive dimorphic sequences, which define the two allelic families of MSP2, 3D7 and FC27, respectively. As it is abundantly expressed on the merozoite surface [12], MSP2 is a potential target of antibodies induced by infection or vaccination. The majority of individuals living in regions where there is intense transmission of *P. falciparum* develop high titres of anti-MSP2 antibodies in response to repeated infections [13]. Much of the natural anti-MSP2 antibody response is directed against epitopes in the central variable region of the molecule as well as the dimorphic regions [14]. Several sero-epidemiological studies, but not all [15], have shown an association between antibody responses to MSP2 and resistance to infection or disease [16], [17], [18], [19]. MSP2-specific antibodies in immune individuals are predominantly of the cytophilic IgG3 subclass [14], [17], [18], [20].

Evidence that MSP2 has potential as a component of a malaria vaccine came from the testing of the Combination B vaccine in Papua New Guinea. This vaccine contained three recombinant malaria proteins, all expressed in *E. coli*: full-length 3D7 MSP2, a small N-terminal fragment of MSP1, and a large C-terminal fragment of the ring-infected erythrocyte surface antigen. This three component vaccine was adjuvanted with Montanide® ISA 720 (ISA 720) [21], [22], [23]. The development of Combination B culminated in a Phase 1/2b double-blind randomized placebo-controlled trial in 120 children 5–9 years of age living in the Wosera region of the East Sepik Province of Papua New Guinea [23]. One half of the subjects in this trial (60 individuals) were drug-treated to eliminate existing parasitemias before vaccination. The incidence of malaria parasitemia in these drug-treated individuals was very low, a factor that impeded assessment of vaccine efficacy in this group. In contrast, a significant reduction in parasitemia was observed among the 30 vaccinated individuals who were not drug-treated, with mean parasitemia reduced by 62% (p = 0.024). Analysis of the *msp2* genotypes in breakthrough parasite populations demonstrated that among vaccinees in both the drug-treated and untreated groups, there was a lower prevalence of infection with parasites carrying the 3D7 allelic form of MSP2 (corresponding to that in the vaccine). Furthermore, over the 12-month follow-up, there was a higher incidence of morbidity associated with *P. falciparum* infections of the FC27 *msp2* genotype in the vaccine group [23]. This indicated that activity of the Combination B vaccine was at least partially attributable to the MSP2 component of the vaccine. Furthermore, the results suggested that the vaccine may exert a selective pressure on the parasite population by inducing immune responses that are more active against parasites expressing forms of MSP2 belonging to the 3D7 dimorphic family than against parasites expressing forms of MSP2 belonging to the FC27 dimorphic family. This finding indicated that any further testing of a MSP2 vaccine should include antigens representative of both families of MSP2 alleles, which have been found in all infected populations examined [24].

Therefore we undertook a dose-escalating, double-blinded, placebo-controlled Phase 1 trial in healthy, malaria-naïve adults of a new MSP2 vaccine (MSP2-C1) containing the 3D7 and FC27 forms of MSP2, representative of the two families of MSP2 alleles. Equal amounts of the two antigens were formulated in a water-in-oil emulsion with ISA 720. Placebo subjects received saline emulsified in ISA 720. The aims of the study were to test safety and immunogenicity of this new vaccine.

## Materials and Methods

### Ethics Statement

The protocol for this trial and supporting CONSORT checklist are available as supporting information; see Checklist (Checklist S1) and Protocol (Protocol S1) with Amendments (Protocol S2, Protocol S3 and Protocol S4). The study was approved by the Queensland Institute of Medical Research Human Research Ethics Committee (QIMR-HREC) and by the Western Institutional Review Board, which is the designated research ethics board for PATH MVI, the sponsor of the trial. The study was conducted in accordance with the Declaration of Helsinki principles for the conduct of clinical trials and the International Committee of Harmonization Good Clinical Practice Guidelines as recognized by the Australian Therapeutic Goods Administration (TGA; (www.tga.gov.au/docs/pdf/euguide/ich/ich13595.pdf). The trial was conducted with Regulatory oversight by the TGA under the CTN scheme, and registered at the Australian and New Zealand Clinical Trials registry (www.anzctr.org.au/ACTRN12607000552482.aspx).

### Vaccine preparation

The two antigens in MSP2-C1 were manufactured at GroPep Pty Ltd (Adelaide, Australia). Briefly, both 3D7 and FC27 MSP2 were expressed in *E. coli* from synthetic genes cloned into the *E. coli* expression plasmid pET22b (GenBank Accession numbers: JN248383 and JN248384 respectively). Each recombinant protein had a 6-histidine C-terminal tag to allow purification by metal-chelate chromatography. The two recombinant proteins (*Ec*MSP2-3D7 and *Ec*MSP2-FC27) were purified from *E. coli* lysates by a combination of chelating, anion-exchange and reverse-phase chromatography. The purification processes were designed to separate monomeric MSP2 from non-product related components (e.g. *E. coli* proteins, DNA and endotoxin), as well as MSP2 aggregates or fragments. Both recombinant forms of MSP2 have a propensity to form amyloid-like fibrils [25] but this was inhibited by storing the purified antigens at -80°C in 10 mM acetic acid. *Ec*MSP2-3D7 and *Ec*MSP2-FC27 were shown by analytical reverse-phase chromatography to be >95% and >93% monomer, respectively. On SDS-PAGE a single species was detected in each of the protein preparations but the electrophoretic mobility of *Ec*MSP2-3D7 and *Ec*MSP2-FC27 differed slightly, thus allowing the two proteins to be distinguished when extracted from the formulated vaccine. Equal amounts of the two antigens were formulated in the experimental adjuvant ISA 720 (SEPPIC Inc) at CSL Ltd (Melbourne, Australia). A water-in-oil emulsion, with a droplet size of ∼1 micron, was generated by homogenization. Glycine (final concentration 250 mM) was added to the aqueous phase prior to homogenization because this has been shown to minimise modifications that can occur in ISA 720 emulsions [26]. The individual antigens stored at −80°C and the antigens in the formulated vaccine, stored at 4°C were shown to be stable for 24 and 18 months, respectively.

### Study design

The study was designed as a dose-escalating, placebo-controlled Phase 1a trial to assess the safety and immunogenicity of three dosages of MSP2-C1/ISA720. The study was planned to include 45 volunteers to be vaccinated in three cohorts of 15 (10, 40, or 80 µg of MSP2-C1 formulated in ISA 720); volunteers in each cohort were randomized to receive vaccine (n = 12) or ISA 720 emulsion as a control (n = 3), with a dosing interval of 12 weeks planned between each immunization. The three cohorts were screened and enrolled consecutively. For cohorts two and three, it was planned that immunizations be staggered, such that the first five volunteers of both cohorts be vaccinated four weeks before the remaining 10. All study staff including the volunteers, investigators, individuals responsible for the evaluation of study subjects and laboratory personnel were blinded to the randomization scheme except for the study pharmacist (or designee) who prepared the vaccine dose for administration. The volumes of vaccine and ISA 720 Control were identical for each group. Code-break envelopes for individual participants were provided to account for the contingency that the Investigator needed to break the code for an individual subject.

### Participants

A total of 45 healthy, malaria naïve male and female volunteers, aged between 18 and 45 years were recruited from a database of healthy volunteers maintained by Q-Pharm, or by advertisement to students of The University of Queensland or to the general community. A screening medical history, a physical examination, and laboratory testing were performed after written informed consent was obtained. Details of the inclusion and exclusion criteria are available in the Protocol in the supplemental material.

### Assessment of safety

Volunteers were observed for at least 30 minutes after each vaccination, contacted by phone on day 1 and evaluated at the study site on days 3, 7 and 14 after every immunization. At each visit, the volunteers underwent an abbreviated history and physical examination, including examination of injection site, emphasising examination of any acute complaints. Local reactions at the injection site were evaluated according to Brighton collaboration definitions, (www.brightoncollaboration.org). Safety was also assessed by serial laboratory evaluations of clinical chemistry and hematology on blood samples collected on days 3, 14 and 56 days after immunization. After the detection of creatine phosphokinase elevations (CPK) in two volunteers following the first dose of 40 µg MSP1-C1, a specific history indicating symptoms of muscle damage (exercise history, symptoms of myositis) was solicited in volunteers. Progress of the trial and review of its safe conduct was overseen by a safety monitoring committee, comprising three independent medical experts.

### Assessment of Immunogenicity

For evaluation of immunogenicity, blood samples were collected on Day 0 prior to receipt of the first vaccination, one month after each immunization, and at the end of study on Day 360 for assessment of antibody responses by ELISA, immunofluorescence assays (IFA) with fixed *P. falciparum* schizonts, *in vitro* growth inhibition assays (GIA), Western blotting of parasite protein extracts, and antibody-dependant cellular inhibition assay (ADCI). Peripheral blood mononuclear cells (PBMC) were isolated from heparinised blood samples, washed in PBS and immediately used for lymphocyte stimulation assays.

ELISAs were performed using established methods [27], [28]. 96-well plates (Maxisorp, Nunc, Roskilde, Denmark) were coated with recombinant proteins at 1 µg/ml in PBS overnight at 4°C. Plates were washed and blocked with 0.1% casein in PBS with 0.05% Tween 20 (PBS-T) for 2 hours. After washing, serum samples (100 µl/well in duplicate), diluted in PBS-T plus 0.1% casein, were incubated for two hours. Plates were washed and incubated for one hour with HRP-conjugated anti-human IgG at 1/5000 (Chemicon, Melbourne, Australia) in PBS-T plus 0.1% casein. After washing, colour was developed by adding azino-bis(3-ethylbenthiazoline-6-sulfonic acid) liquid substrate system (Sigma-Aldrich, Sydney; stopped with 1% SDS after 15 mins) and the absorbance was read by spectrophotometry. All washes were performed with PBS-T, and all incubations were at room temperature. Positive and negative controls were included on all plates to enable standardisation. Serum samples were tested at 1/100 and 1/500 dilution; only results from testing at the 1/100 dilution are reported as no major differences in the pattern of reactivity were found when testing samples at 1/500. All available serum samples were tested for IgG responses to recombinant 3D7 and FC27 MSP2, the two vaccine antigens. Samples tested were: Cohort 1 – Day 0, 28, 84, 112, 168, 196, and 336; Cohort 2 – Day 0, 28, 84, 112, 168, 336 (note that day 196 was not available for this cohort). All samples were randomized and coded for testing in a blinded manner.

For Western blot and immunofluorecent antibody assays (IFA) schizont-stage isolates of the *P. falciparum* strains 3D7 and D10 [a clone of the FC27 parasite line] were prepared from parasites cultured in RPMI-HEPES with 0.5% Albumax [4]. A subset of samples (n = 3) for testing in western blots and IFAs were selected on the basis that they were highly reactive by ELISA (in the top half of responses) at day 112. For IFA, parasites were washed and smeared onto glass slides and fixed with methanol/acetone (10∶90, v∶v). Smears were blocked with 0.1% casein in PBS, incubated with cohort samples (diluted 1∶1000), then Alexa-Fluor 488-conjugated anti-human IgG. Slides were cover-slipped and examined by fluorescence and light microscopy. For Western blots, parasite proteins were extracted into 1% Triton X100 (TX100) in PBS with protease inhibitors (Roche), separated by SDS-PAGE and transferred onto PVDF membranes. Membranes were blocked with 0.1% casein in PBS, incubated with cohort samples diluted 1/500 in PBS with 0.1% casein, and then with HRP-conjugated anti-human IgG. IgG-binding was determined by chemiluminescence.

PBMC for lymphocyte stimulation assays were prepared in Hepes-buffered RPMI-1640 with, 100 IU/mL Penicillin-Streptomycin (GIBCO BRL, Invitrogen), and heat-inactivated autologous serum in 96 well flat-bottomed plates in a volume of 200 µl/well. Positive control cultures were established with 10% serum and the mitogen phytohaemagglutinin (PHA; 2.2 µg/ml). Antigen-specific cultures were established with heat-inactivated autologous serum at a concentration of 20% and each antigen individually at 1 and 10 µg/ml. Plates were incubated (six days, 37°C, humidified 5% CO2) before labelling (25 µl ^3^H thymidine, 40 µCi/ml, 16–18 hours) and harvested onto filter mats using a PerkinElmer FilterMate harvester. Incorporated ^3^H-thymidine was determined using a PerkinElmer MicroBeta TriLux 1450 LSC Luminescence counter. Stimulation indices (SI) were calculated relative to control wells to which no recombinant antigen had been added. For inclusion of data, replicate values of thymidine incorporation were required to be within 150% of each other, and replicate unstimulated and mitogen-stimulated values to be consistent.

Two growth inhibition assays (GIAs) were used to assess the ability of induced anti-MSP2 antibodies to inhibit parasite replication in vitro. In one assay serum samples from day 0 and day 112 were tested in a two-cycle flow cytometry-based assay [27], [29] for all subjects at 1/10 and 1/5 dilution against 3D7 and D10 (FC27) parasites. Randomized and coded serum samples were filter sterilized before testing. The *P. falciparum* lines 3D7 and D10 were cultured *in vitro* [4] and synchronised by alternate day re-suspension in 5% D-sorbitol (Sigma, St Louis, MO, USA) in water. Growth assays were performed over two cycles of parasite replication and parasitemias were measured using flow cytometry (FacsCalibur, Becton, Dickinson, Franklin Lakes, NJ). Assays were set-up with mature trophozoites and schizonts, at 1% hematocrit and 0.3% starting parasitemia (50 µl/well) in 96 well U-bottom culture plates (Becton Dickinson, Franklin Lakes, USA) with serum samples from cohort members. On all plates, PBS and samples from non-exposed Australian residents were included as a non-inhibitory controls and 1F9 anti-AMA1 monoclonal antibody [30] was included as an inhibitory control. Parasite growth for each sample is expressed relative to plate PBS controls. All samples were tested in duplicate in two separate assays.

GIA was also assessed using IgG fractions isolated from serum samples collected from 10 subjects on days 0 and 112.; all subjects were positive for antibodies to MSP2 by ELISA on day 112. IgG from serum samples was purified using protein G-Sepharose columns, dialysed, and then concentrated to 20 mg/ml. IgG samples, at a final concentration of 10 mg/mL, were incubated with the 3D7 *P. falciparum* blood-stage parasites for ∼40 hours. Parasite growth was then determined by colorimetric quantification of *P. falciparum* lactate dehydrogenase, [31].

The effect of purified IgG on parasite growth, measured by antibody-dependant cellular inhibition (ADCI) was performed as described previously [32]. IgG samples purified from the day 0 and day 112 of 10 subjects (as described above) were tested at a final concentration of 1 mg/ml for ADCI activity against the 3D7 and K1 parasite lines, expressing the 3D7 and FC27 *msp2* alleles, respectively. Isolates were obtained from MR4 and identity was confirmed by genotyping. Potential contamination of cultures with Mycoplasma was excluded by PCR testing. PBMC from healthy and malaria naïve blood donors were separated by Ficoll-Hypaque and then aliquoted at a final concentration of 25×10^6^ cells/ml in cryopreservation medium and stored in liquid phase of liquid nitrogen. Aliquots were kept in liquid nitrogen until use. Identification and enumeration of the monocyte sub-population was assessed by flow cytometry using anti-CD14-PE (mAb clone RMO2, a murine IgG2a, that emits a yellow colour at 575 nm), and anti-CD16-PC5 (mAb clone 3G8, a murine IgG1 that emits in dark red-brownish at 670 nm); both from Beckman Coulter. A single batch of monocytes was used for all experiments performed. Assays were performed in 96-well culture plates containing 2×10^5^ monocytes per well with 100 µl of an asynchronous culture of either 3D7 or K1 *P. falciparum*-infected red blood cells at 0.5% parasitemia and 2.5% hematocrit. Cultures were incubated for 72 hours and the number of infected erythrocytes per well was determined by flow cytometry using double-staining with hydroethidine and thiazole orange dyes [33]. IgG purified from African adults from Ivory Coast employed previously to passively transfer protection, and IgG purified from non-exposed donors were used as positive and negative controls respectively. Specific growth inhibition (SGI) was calculated as SGI = 100×[1-(percent parasitemia with MN and test sample/percent parasitemia with test sample)/(percent parasitemia with MN and control IgG/percent parasitemia with control IgG)]. As previously described, the threshold of positivity in this assay is ≥30% SGI. To take into account experimental variations results are reported as Adjusted-SGI relative to the positive reference control employed in each ADCI plate.

### Statistical methods

#### Sample Size

A group size of 12 volunteers per dose, with 9 participants receiving the MSP2-C1/ISA720 malaria vaccine and 3 per dose cohort receiving ISA 720 Control vaccine was selected, as it was powered to provide sufficient safety data on an adult population living in an area without malaria. It was calculated to have a power of 80% for detecting one or more serious or severe adverse events that occurred with a probability of 0.15 per volunteer.

#### Statistical Analysis

The incidence of adverse events (AEs) and serious adverse events (SAEs) were tabulated by dose and treatment group, with the pooled ISA 720 Control patients representing one group. It was planned to compare the incidence in each treatment group with that in the pooled ISA 720 Control group using Fisher's exact test (correcting for multiple comparisons across AEs using Holm's method [34]). The overall number and percentage of volunteers with at least one AE and SAE were tabulated after each vaccination and over the entire study period. The incidence, intensity, and relatedness to vaccination of individual symptoms were calculated for each dose and study arm. The incidence, intensity and relatedness to vaccination were calculated for each treatment group. Safety analyses were based on intention to treat data selection.

Antibody levels between study groups at each time point were compared using Mann-Whitney U tests. Wilcoxon signed rank sum tests were used to compare the difference in antibody levels and ADCI activity between day 0 and other time points. The correlation between MSP-2-3D7 and MSP-2-FC27 responses at day 112 was assessed using Spearmans rho. One subject (subject 4) withdrew after the baseline bleed and was thus excluded from analysis. All other data points were used in the analysis. All p values<0.1 are shown. Analysis was performed using GraphPad Prism, and SPSS software packages.

## Results

### Participants

**Figure 1:**
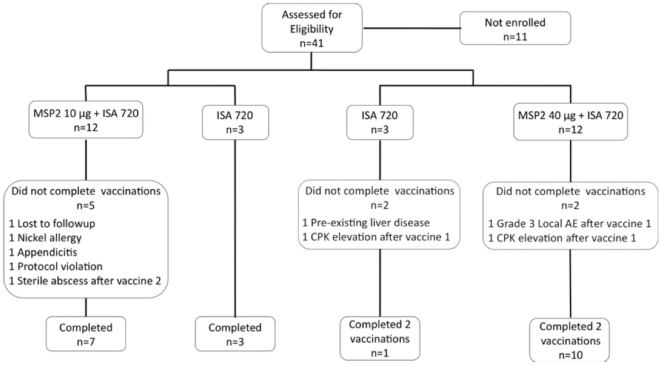
Participant Flow.

Participants were recruited at the trial site, Q-Pharm Pty Ltd, Brisbane, QLD, Australia from November 2007 until February 2008. Of 41 potential study subjects screened for enrolment in cohorts one and two, 30 were deemed eligible and enrolled (Figure 1). Reasons for exclusion included declining to consent (n = 6), possible past malaria exposure (n = 1), and incompatible chronic medications (n = 1). Table 1 shows the demographics of volunteers per randomised group. The mean age was 26 (range 19–37 years); 25 of 30 subjects were of Caucasian background.

**Table 1:** Study subjects.

		Cohort 1MSP2 10 µg+ISA 720(n = 12)	Cohort 2MSP2 40 µg+ISA 720(n = 12)	Pooled Control ISA 720(n = 6)
Race	Caucasian	11	10	4
	Asian	1	0	1
	South Asian	0	1	1
	Latin American	0	1	0
Age	Mean (SD)	24.2 (6.6)	28.1 (7.1)	25.7 (5.3)
	Range	18–39	19–39	20–33
Sex	Male/Female	4/8	4/8	4/2

### Participant flow

Ten of 15 subjects in cohort 1 received all three immunizations. Five subjects, all in the vaccine arm of cohort 1 did not receive all three doses of vaccine (Figure 1, Table 2). Four of these five were excluded for reasons judged to be unrelated to vaccination. These included loss to follow-up (n = 1; after first immunization); bilateral ear lobe dermatitis suspected to be due to Nickel allergy (n = 1; after second immunization); appendectomy (n = 1; after second immunization); received Human Papilloma Virus immunization in the interval between screening and enrolment (n = 1). The fifth volunteer was withdrawn from the study after developing a sterile abscess after the second dose of vaccine (see below).

**Table 2:** Subjects not completing study procedures.

Group	Vaccine doses received	Reason
MSP2 10 µg+ISA 720	1	Lost to Follow-up
	1	Jewellery allergy (Concern re Nickel allergy)
	1	Protocol violation
	2	Appendicitis
	2	Sterile abscess at injection site
MSP2 40 µg+ISA 720	1	Grade 3 Local AE after vaccination
	1	Withdrawn from further vaccination because of exercise-induced CPK elevation
Pooled Control ISA 720	1	Pre-existing fluctuating LFT abnormality discovered
	1	Withdrawn from further vaccination because of exercise-induced CPK elevation

Eleven of the 15 enrolled subjects in cohort 2 received two immunizations. Four volunteers received one immunization, 3 for reasons judged not to be related to vaccination (n = 1: discovery of pre-existing hepatic condition, the ISA 720 control group; n = 2: significant, exercise-related transient CPK elevation, one subject in each treatment arm). One subject in the vaccine arm of cohort 2 was withdrawn from receipt of further doses of vaccine after developing a Grade 3 local reaction (see below) but was followed for safety for the duration of the study. The investigators, in consultation with the Safety Monitoring Committee decided not to proceed with immunizing Cohort 3 subjects due to two observed severe local reactions that had occurred in Cohort 1 and 2 subjects. Due to a failed *in vivo* potency test of the stored formulated vaccine at 13 months, Cohort 2 subjects did not receive their third scheduled immunization.

### Safety and reactogenicity

No serious adverse events occurred that were definitely, probably, or possibly related to immunization. As noted above, one subject (cohort 2, ISA 720 control) was discovered to have pre-existing fluctuating derangements in liver function tests. A further two volunteers (1 subject in each arm of cohort 2) were found to have transient elevations in ALT and AST levels, as well as significantly elevated CPK levels. These were attributed in both cases to vigorous physical exercise that they had undertaken in the days immediately preceding the blood collections. No other clinically relevant changes in vital signs or laboratory values were reported throughout the study. Volunteers in both Cohort 1 and Cohort 2 experienced local injection site reactions. The most commonly reported reaction was injection site pain, with 29 of 30 subjects reporting at least transient mild to moderate pain (Table 3). These reactions were sufficiently severe to require the withdrawal of two volunteers from further vaccination. No relationship was discernable between the likelihood of local reaction and the dose of vaccine administered or whether it was the first or subsequent immunization. One subject in the 10 µg vaccine cohort developed an abscess measuring 3×2 cm 3 days after the second immunization, and one subject in the 40 µg vaccine group developed grade 3 pain, swelling and erythema, accompanied by moderate induration beginning on the day of the first immunization.

**Table 3:** Local Reactions.

		Cohort 1MSP2 10 µg+ISA 720(n = 12)	Cohort 2MSP2 40 µg+ISA 720(n = 12)	ControlISA 720 10 µg(n = 3)	ControlISA 720 40 µg(n = 3)	Total
Pain						
	Mild	12	7	2	2	23
	Moderate	0	4	1	0	5
	Severe	0	1††	0	0	1
Swelling						
	Mild	3	1	0	0	4
	Moderate	1	2	1	0	4
	Severe	0	1††	0	0	1
Erythema						
	Mild	0	0	1	0	1
	Moderate	0	2	0	0	2
	Severe	1	1††	0	0	2
Induration						
	Mild		1	1		2
	Moderate		2			2
	Severe					
Nodule						
	Mild		1			1
	Moderate					
	Severe					
Sterile Abscess		1†				

† 3×2 cm abscess beginning three days after second immunization; subject withdrawn from further doses of vaccine; abscess resolved spontaneously.

†† Subject developed severe pain, swelling and erythema, accompanied by moderate induration beginning on the day of the first immunization; subject withdrawn from further doses of vaccine; adverse events resolved spontaneously.

### Humoral immune response

**Figure 2:**
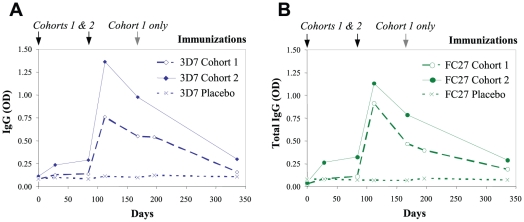
Antibodies to MSP2 recombinant proteins were induced by immunization. Serum IgG binding to recombinant MSP2 representing the 3D7 (A) and FC27 (B) alleles was measured by ELISA at all available time points from cohort 1 and cohort 2, comparing those who received active vaccine versus placebo. Results show the median at each time point; placebos from cohort 1 and 2 were combined. P<0.01 comparing day 0 versus day 112 IgG levels for cohort 1 and 2, and for 3D7 and FC27 (excluding placebo recipients); p<0.01 comparing placebo versus active vaccine recipients at day 112 for 3D7 and FC27. Immunizations were given at day 0, day 84, and day 168 for cohort 1 (20 ug/dose), and day 0 and day 84 for cohort 2 (40 µg/dose).

Immunization induced significant IgG responses to the FC27 and 3D7 isoforms of MSP2 in both cohorts 1 and 2 (Figure 2; Table 4). One month after the first immunization (day 28) sera from both cohorts 1 and 2 had higher antibody levels against 3D7 MSP2 (P = 0.062 and P = 0.008 in cohorts 1 and 2, respectively) and FC27 MSP2 (P = 0.004 and P = 0.003, respectively) compared to day 0. At day 112 (3 months after day 0, and 1 month after dose 2) antibody responses peaked and were significantly higher compared to day 0 for both antigens (P≤0.003). After day 112, antibody responses waned; although responses were higher at day 336 compared to day 0 for both antigens (P≤0.01), they were comparable to day 28 levels (P>0.11). Only subjects in Cohort 1 received a third dose of vaccine at day 168 and interestingly, one month after this third dose (day 196) there was limited increase in IgG to both 3D7 and FC27 MSP2 (P = 0.11 and P = 0.075, respectively, Figure 2; Table 4), and responses were lower than those seen at day 112 (P≤0.021). Antibodies at day 112 to 3D7 and FC27 MSP2 were highly correlated (r = 0.934, P<0.001).

**Table 4:** IgG responses for MSP2-3D7 and MSP2-FC27 according to study day and cohort.

	Day 0	Day 28	Day 84	Day 112	Day 168	Day 196	Day 336
Antibody and dilution	Median (range) No. Positive (%)^a^	Median (range) No. Positive (%)^a^	Median (range) No. Positive (%)^a^	Median (range) No. Positive (%)^a^	Median (range) No. Positive (%)^a^	Median (range) No. Positive (%)^a^	Median (range) No. Positive (%)^a^
**MSP-2-3D7 IgG**							
**Cohort 1**	0.080 (0.033–0.203)	0.125 (0.030–0.732)	0.138 (0.035–0.504)	0.757 (0.123–1.490)**	0.548 (0.068–1.368)*	0.539 (0.071–1.277)	0.156 (0.048–0.771)
	2/11 (18.2)	4/11 (36.4)	5/11 (45.5)	9/11 (81.8)	8/11 (72.7)	8/11 (72.7)	5/11 (45.5)
**Cohort 2**	0.111 (0.044–0.402)	0.232 (0.087–0.641)†	0.289 (0.072–0.772)*	1.360 (0.129–1.649)***	0.974 (0.116–1.544)***	.	0.298 (0.123–1.169)*
	1/12 (8.3)	8/12 (66.7)	9/12 (75)	11/12 (91.7)	11/12 (91.7)	.	11/12 (91.7)
**Placebo**	0.081 (0.067–0.483)	0.098 (0.059–0.700)	0.081 (0.048–0.495)	0.110 (0.033–0.542)	0.098 (0.040–0.517)	0.12 (0.052–0.529)	0.105 (0.046–0.550)
	1/6 (16.7)	1/6 (16.7)	1/6 (16.7)	1/6 (16.7)	1/6 (16.7)	4/6 (66.7)	1/6 (16.7)
**MSP-2-Fc27 IgG**							
**Cohort 1**	0.028 (0.021–0.094)**	0.082 (0.031–0.567)†	0.110 (0.036–0.460)	0.915 (0.146–1.319)**	0.467 (0.067–1.298)*	0.397 (0.057–1.176)*	0.189 (0.035–0.767)
	1/11 (9.1)	4/11 (36.4)	8/11 (72.7)	11/11 (100)	10/11 (90.9)	10/11 (90.9)	8/11 (72.7)
**Cohort 2**	0.043 (0.027–0.219)	0.264 (0.041–0.469)	0.323 (0.059–0.718)*	1.132 (0.086–1.552)***	0.785 (0.067–1.363)**	.	0.287 (0.084–1.146)†
	3/12 (25)	10/12 (83.3)	10/12 (83.3)	11/12 (91.7)	11/12 (91.7)	.	11/12 (91.7)
**Placebo**	0.076 (0.049–0.266)	0.081 (0.051–0.292)	0.071 (0.043–0.282)	0.069 (0.047–0.322)	0.066 (0.045–0.265)	0.087 (0.072–0.101)	0.071 (0.067–0.238)
	2/6 (33.3)	2/6 (33.3)	1/6 (16.7)	2/6 (33.3)	1/6 (16.7)	4/6 (66.7)	1/6 (16.7)

a Positives were defined as those with an OD greater than median +3 median absolute deviations calculated from antibody data from Day 0 bleeds (all groups combined).

† *P*<0.1,

* *P*<0.05,

** *P*<0.01,

*** *P*≤0.001.

*P*-values represent comparisons of Cohort 1 and Cohort 2 with Placebo group as assessed using Mann-Whitney *U* test.

As anticipated, IgG levels to both 3D7 and FC27 MSP2 were significantly higher in the active than in the placebo group (P≤0.003) at day 112. Although total IgG responses waned after day 112, there was still evidence of higher antibody levels in the active group at day 196 and day 336 (Table 4). Median 3D7 MSP2 IgG levels were consistently higher in cohort 2 compared to cohort 1 at all time points (Figure 2; Table 4), although this did not reach statistical significance except at day 168 for which there was some evidence of increased 3D7 MSP2 responses in cohort 2 compared to cohort 1 (P = 0.045). For FC27 MSP2, levels of IgG were generally higher in cohort 2 compared to cohort 1 at day 28 (P = 0.049), day 84 (P = 0.036), day 112 (P = 0.14) and day 168 (P = 0.045) (median [range] values shown in Table 4).

### Recognition of native MSP2 by serum antibodies

**Figure 3:**
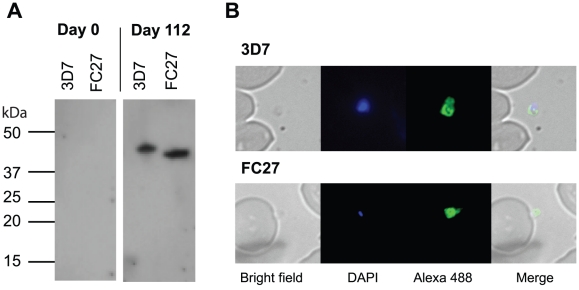
Labelling of native proteins of *P. falciparum* strains 3D7 and D10 (a clone of FC27) by Western blot (A) and immunofluorescence microscopy (B). **A**. Serum antibodies from a representative subject collected at day 112 post-immunization bound to MSP2 present in schizont protein extracts. Serum antibodies from the same subject collected at baseline (day 0) did not react with parasite proteins. **B**. Serum antibodies from a representative subject collected at day 112 post-immunization labelled merozoites.

Selected samples collected at day 112 that had high IgG reactivity to recombinant proteins by ELISA were tested for antibody reactivity with native proteins. In Western blots, ELISA-positive day 112 samples clearly labelled bands corresponding to MSP2 in schizont protein extracts from 3D7 and D10 parasites (Figure 3A). There was no reactivity observed with day 0 samples collected from the same individuals. ELISA-positive day 112 samples also labelled merozoites by indirect immunfluorescence assays using fixed smears of schizont-stage parasites of 3D7 and D10 lines (Figure 3B). There was no labelling of merozoites by day 0 samples collected from the same individuals.

### Antibody inhibition of *P. falciparum* growth *in vitro*

#### GIA

**Figure 4:**
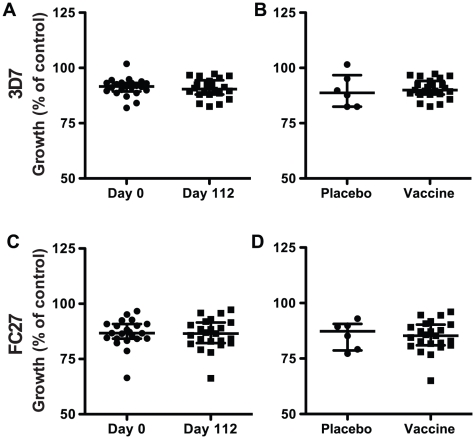
Immunization with MSP2 did not induce growth-inhibitory antibodies. Serum samples collected from all subjects at day 0 and day 112 post-immunization were tested in growth-inhibition assays over 2 cycles of erythrocyte invasion using 3D7 (A, B) and FC27 (C, D) parasites. There was no significant difference in parasite growth in the presence of samples comparing day 0 versus day 112 (A, C; placebo recipients excluded) or active vaccine versus placebo (B, C; day 112 samples only). The median (±interquartile range) is indicated by a horizontal bar.

There was no evidence of GIA activity induced by immunization with the active vaccine when samples were tested at 1/10 and 1/5 dilutions using a FACS-based two-cycle assay (Figure 4). No significant differences were seen when comparing day 112 versus day 0, active vaccine versus placebo at day 112, or cohort 1 versus cohort 2 at day 112. The range of inhibitory activity was very similar for day 0 versus day 112 samples, and for active vaccine versus placebo among day 112 samples. In addition, purified IgG from day 0 and day 112 samples collected from 10 subjects was tested at a final concentration of 10 mg/ml in a one-cycle assay using pLDH activity to measure parasite growth. Again, there was no evidence of GIA activity against 3D7 parasites comparing active vaccine versus placebo (data not shown), even though all day 112 samples were positive for antibodies to MSP2 by ELISA.

#### ADCI

**Figure 5:**
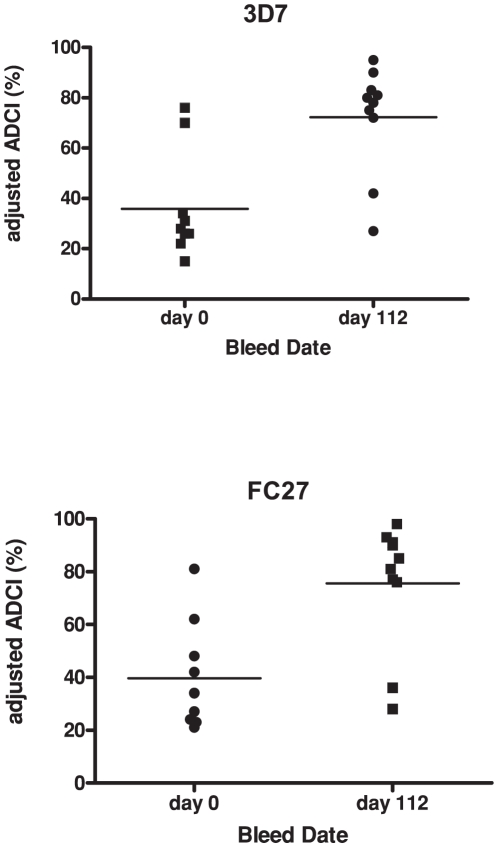
Immunization with MSP2 induced antibody-dependent cellular inhibition activity against P. falciparum. IgG was isolated from serum samples collected at day 0 and day 112 post-immunization from 10 subjects and tested in for ADCI activity using 3D7 parasites or K1 parasites. Results are expressed as inhibition relative to control (adjusted ADCI, %). The median is indicated by a horizontal bar. There was a statistically significant difference in ADCI activity between day 0 and day 112 samples for assays using 3D7 and K1 parasites (p = 0.0108 and p = 0.0059, respectively; Wilcoxon matched pairs test). All samples were positive for antibodies to recombinant MSP2 by ELISA for day 112 samples; no samples from placebo recipients were included.

Purified IgG was prepared from day 0 and day 112 serum samples from 10 individuals who had the highest MSP2 IgG levels by ELISA post-immunization (5 subjects each from cohort 1 and 2). These blinded pre and post immunization samples were tested for ADCI activity against 3D7 and K1 parasites, representing the 3D7 and FC27 MSP2 allelic families, respectively. ADCI activity was significantly higher among day 112 samples compared to day 0 for both the 3D7 and K1 parasite lines (P = 0.012 and P = 0.006, respectively; Figure 5). For 3D7 parasites, median adjusted inhibition was 29.5% for day 0 versus 79.0% for day 112. For K1 parasites, median adjusted inhibition was 34.0% for day 0 versus 83.0% for day 112. There was a strong correlation between ADCI activity for 3D7 versus K1 parasites (r = 0.926; P<0.001). Of note, two day 0 samples had substantial ADCI activity (70% and 76% for 3D7; 62% and 81% for K1), and two day 112 samples had relatively low activity (27% and 42% for 3D7; 28% and 36% for K1). In exploratory assays these four samples were retested in a blinded manner against 3D7, with the results suggesting that a laboratory error had resulted in an inversion of the day 0 and day 112 data for these four samples (see Supplementary Information Table S1).

### Cellular immune response

Interpretation of assays of proliferation of freshly collected and isolated PBMC was hampered by technical problems that resulted in poor agreement between replicate samples, both in control wells containing unstimulated cells and in wells containing vaccine antigen. Nevertheless, the overall data suggested that there was a cellular immune response to 3D7 and FC27 antigens induced in a proportion of volunteers (Supplemental material, Figure S1); stimulation indices at some post-vaccination time points were significantly higher than day 0.

## Discussion

This is the first clinical trial of a malaria vaccine containing two isoforms of MSP2 that represent the two families of *msp2* alleles in *P. falciparum*. Although the vaccine was immunogenic, unacceptable, frequent and occasionally severe injection site adverse events were observed in subjects in the two lower dose cohorts. Because of this unacceptable reactogenicity, a decision was made in consultation with the Safety Monitoring Committee not to proceed with vaccination of subjects in the third and highest dose cohort. Further clinical evaluation of MSP2-C1 will therefore require that it be formulated with an alternative adjuvant.

MSP2-C1 formulated in ISA 720 induced significant antibody responses to both forms of MSP2 in the majority of subjects receiving the vaccine, and an increased antigen dose resulted in higher antibody responses. Furthermore, antibodies induced by immunization recognized native MSP2 by Western blot of parasite protein extracts and IFA of whole parasites. When the potential functional activity of the induced anti-MSP2 antibodies was evaluated, no *in vitro* GIA was observed. However, significant ADCI activity against parasites of both MSP2 types was seen, indicating potentially important functional activity of the vaccine-induced antibodies. These findings provide further support for MSP2 as a blood stage vaccine candidate. This is in addition to sero-epidemiologic evidence for a role for MSP2 in acquired immunity [16], [17], [18], [19], and evidence of protective efficacy of the Phase 2 trial of the MSP2-containing vaccine, Combination B [23].

Although some anti-MSP2 antibodies have been reported to have direct *in vitro* growth-inhibitory activity [35], [36], and there is indirect evidence that naturally occurring antibodies to MSP2 inhibit merozoite invasion [37], our results are consistent with other studies that have shown that naturally-acquired antibodies to MSP2 mediate ADCI but lack growth-inhibitory activity [38]. The reasons for the lack of direct inhibitory activity of human MSP2 antibodies are unclear, as MSP2 is assumed to have an essential role in the attachment and invasion process. Possibly, the antibodies induced by immunizing with recombinant MSP2 do not have the correct fine specificity or a sufficiently high avidity to inhibit invasion directly.

The functional antibody assays were performed on day 112 samples, which coincided with the peak antibody response, four weeks after the second immunization. The antibody responses decayed markedly beyond day 112. Among subjects in cohort 1 who received a third immunization at day 196, only a slight boost in the response was observed. In view of the changes in antibody responses over the course of the trial, it is interesting to note that in the PNG Phase 2b study with Combination B, partial protection of the children was achieved by giving only two immunizations four weeks apart with the ISA 720-formulated vaccine. It is important to note that the limited number of participants who received the intended vaccine schedule, limits the interpretation of the antibody responses induced in the study.

Although the GIA has been widely used to assess antibody responses to asexual blood-stage vaccine candidates, evidence that activity in this assay correlates with protective immunity, and is predictive of vaccine efficacy is limited [29], [39]. There is accumulating evidence that cytophilic antibodies specific for merozoite surface proteins, which are required for ADCI and possibly other Fc receptor-mediated effector mechanisms, are associated with clinical immunity [18], [40], [41] to this and other malaria vaccine candidates [5]. In this study, ADCI activity was seen across a range of volunteer IgG concentrations, an observation in agreement with previous findings [38], [42]. The positive outcome of the ADCI experiments with both the 3D7 and FC27 alleles of MSP2 is in keeping with the ELISA data showing satisfactory antibody responses to both MSP2 alleles. Assessing the role of these different effector mechanisms mediated by cytophilic anti-MSP2 antibodies, and the development of standardized assays for them, will be important for the further development of the MSP2-C1 vaccine and other blood stage vaccine candidates.

In previous human trials of recombinant MSP2, only a single (3D7) form of MSP2, known as Ag1624, was studied. Ag1624 differs from the 3D7 MSP2 used here in that the hexa-His tag was located at the N-terminus rather than the C-terminus, and there were a small number of additional amino acid residues, at both the N- and C-termini of Ag1624, which were derived from the cloning site in the expression plasmid. In the first trial of Ag1624 where it was evaluated in combination with recombinant circumsporozoite protein (CSP) formulated on alum [43], no serious adverse events, and only mild injection site reactions were observed. However, the immune response was suboptimal, and no protection was observed when 5 subjects were experimentally challenged with *P. falciparum* after a third immunization [22].

After immunogenicity studies in animals [44], ISA 720 was chosen as the adjuvant to provide enhanced immune responses to Ag1624 and the other components of the Combination B vaccine (MSP1 and RESA). In two Phase 1 trials conducted in Australia in 1997 to test the safety and immunogenicity of the Combination B/ISA 720 vaccine candidate in healthy Australian volunteers [21], local reactions were common, particularly pain and tenderness on use of the injected muscle beginning shortly after vaccination and lasting a few days. In a smaller number of subjects (10 of 26 in the second study), delayed pain and tenderness developed approximately 10 days after the initial injection. The secondary reactions were mild to moderate in all but one of the subjects who developed severe pain and swelling in the injected thigh 10 days after the dose. When this subject was administered a second dose in the other thigh, the initial local response was mild. However, six weeks later a hard painful mass that limited mobility developed at the second injection site. This resolved over a few weeks. In the challenge trial referred to above [22], where the effect of the Combination B/ISA720 vaccine on *in vivo* parasite growth rate was tested in 12 subjects receiving active vaccine compared to five control subjects who received ISA 720 alone, one of the 12 volunteers had pain and tenderness after the first vaccine dose that was of sufficient severity to preclude a second injection.

Combination B was then assessed in two trials in Papua New Guinea. The first study [45], undertaken in 10 healthy adult males was to assess the safety and immunogenicity. Poor cellular immune responses to MSP2 were observed, with no change in the anti-MSP2 antibody titers from baseline values. Approximately half the subjects had mild pain at the injection site. Subsequently, a Phase 1/2b double-blind randomized placebo-controlled trial was carried out in 120 children 5–9 years of age living in a malaria endemic region of PNG [23], [46]. No serious or severe adverse events occurred in children receiving vaccine or placebo. It was in this study that the protective efficacy of the MSP2 component was identified.

In addition to five trials of MSP2 where ISA 720 has been used as the adjuvant, ISA 720 has been used in 10 Phase 1 studies testing safety and immunogenicity of other malaria vaccine candidates [47], [48], [49], [50], [51], [52], [53], [54], [55], [56] and in three non-malaria Phase 1 clinical trials [57], [58], [59]. As has been observed in the MSP2 trials, formulations of other malaria vaccines with ISA 720 have in general been more reactogenic than those formulated with Alum, with the most common adverse events being local reactogenicity at the injection site. While local reactions were more common in trials where the vaccine was given subcutaneously [48], [52] than when given by the intramuscular route, sterile abscess and moderate to severe local reactions have been reported in studies where ISA 720-formulated vaccines have been administered intramuscularly. It has been suggested that the rather unusual pattern of delayed local reaction that has been reported in volunteers administered ISA 720-formulated vaccines is caused by Arthus-type reactions (type III hypersensitivity) induced by antibodies to the vaccine antigen [51]. However, it has not been possible to confirm this by histological analysis as no biopsies have been taken.

The three non-malaria phase 1 clinical trials where ISA 720 has been used as an adjuvant include a trial of an experimental HIV-1 vaccine [57], a peptide vaccine against Epstein Barr virus (EBV) infection [58], and a melanoma vaccine [59]. The HIV vaccine was highly immunogenic but the reactogenicity in many subjects was severe, with pain & tenderness, induration, erythema, granuloma and sterile abscess formation reported to be mild to severe. The severity of the local reactions was related to antigen dose, the highest dose (1 mg) being much higher than in any other clinical trial of an antigen formulated in ISA 720. Local adverse effects were also more frequent and severe after the second and third doses than after the first dose. In the EBV vaccine study, while the vaccine was well tolerated, mild to moderate injection site reactions occurred in 12 of the 14 subjects. In the melanoma vaccine study [59], mild to moderate local reactions were reported in all patients.

Homogenization is required to generate stable water-in-oil emulsions of antigens formulated with ISA 720 [26]. This procedure contrasts with the ease of generating oil-in-water emulsions, e.g. RTS,S in ASO1 or 2, which can be formulated by simply mixing at the point-of-injection. A consequence of the need to pre-formulate MSP2-C1 in ISA 720 is the need to obtain stability data on the formulated vaccine. This can be problematic because of the extensive assay development that is required at an early stage of vaccine development [26]. Physicochemical analyses on the formulated vaccine and antigens extracted from the emulsion carried out at CSL Ltd Melbourne on preclinical batches of the vaccine prepared for toxicology tests indicated that the emulsion was stable, and the antigens retained their integrity for 18 months (data not shown). The same vaccine batches were tested for immunogenic stability by immunizing mice with three different antigen doses that were below the threshold for a maximum response. The results from a test carried out after 13 months storage of the preclinical batches suggested a loss of immunogenicity (data not shown) and for this reason the vaccine was unavailable for Cohort 2 to receive the third vaccine dose. The same vaccine batches passed a subsequent immunogenicity test, thus highlighting a problem with using *in vivo* potency as a pass-fail criteria at this early stage of vaccine development.

It is apparent from this study and a range of other human clinical trials of recombinant proteins as malaria vaccine candidates that eliciting a strong immune response is likely to be of major importance in inducing vaccine-induced protection, and that selection of an appropriate adjuvant is critical to ensuring that an appropriate balance between reactogenicity and immunogenicity is achieved. For example, it was only when RTS,S was formulated in an oil-in-water emulsion containing the immune stimulants monophosphoryl lipid A and QS21 that this anti-sporozoite vaccine showed protective efficacy [60]. In addition, vaccine-adjuvant formulations must meet stringent requirements including stability and tolerable reactogenicity. Problems with the selection and use of vaccine adjuvants for malaria have been recently reviewed [61]. A significant development has been the establishment of collaborative networks for sharing vaccine adjuvants for high burden tropical disease, including malaria [62], [63].

The rationale for developing MSP2-C1 was the finding in the Combination B trial that protection was ‘strain-specific’. Therefore, antibody responses to the central dimorphic regions of both types of MSP2 would be required to achieve efficacy against most *P. falciparum msp2* genotypes [23]. It was encouraging to find that trial subjects had equally high antibody titers to both components of MSP2-C1 and that these antibodies had ADCI activity against parasites representative of the two MSP2 types. However, sero-epidemiological studies have not clearly established that ‘allele-specific’ anti-MSP2 antibodies are a component of naturally acquired protective antibody responses [27], [28], [64], and some antibodies with ADCI activity appear to recognize an epitope in the conserved C-terminal region of MSP2 [38]. Consequently, it will be important to compare the fine specificity of anti-MSP2 antibody responses induced by vaccination and infection, and further studies are required to determine the relative importance of antibodies to conserved and variable regions of MSP2 in protecting against infection or disease.

## Supporting Information

Checklist S1 CONSORT Checklist
(DOC)

Protocol S1 Trial Protocol
(DOC)

Protocol S2 Revision one of the Protocol
(DOC)

Protocol S3 Revision two of the Protocol
(DOC)

Protocol S4 Revision two of the Protocol
(DOC)

Figure S1 Lymphoproliferative responses to recombinant MSP2 proteins. Peripheral blood mononuclear cells were isolated from blood samples and tested in lymphoproliferation assays for responses to recombinant MSP2 of the 3D7 and FC27 alleles. Results are expressed as the stimulation index (SI), presented as box-and whisker plots showing the median (horizontal line), inter-quartile range (box at each time point for subjects receiving the active versus placebo vaccine. ‘Screen’ indicated the day 0 sample. The numbers for each comparison for are as follows: screen v d28, n = 19; screen v d112, n = 19.
(TIF)

Table S1 Two day 0 samples (marked with an asterisk) had substantial ADCI activity (70% and 76% for 3D7), and two day 112 samples (marked with an asterisk) had relatively low activity (27% and 42% for 3D7). In exploratory assays these four samples were retested in a blinded manner in ADCI assays, together with their matched day 0 or day 112 samples. Results show the adjusted ADCI values from the original assay and the repeat assay. Shown here is one example of two such re-test experiments, both of which yielded consistent results. All assays were performed with the *P. falciparum* 3D7 line using purified IgG.
(DOC)
