# Supplementary material for: A Phase 1 Trial of MSP2-C1, a Blood-Stage Malaria Vaccine Containing 2 Isoforms of MSP2 Formulated with Montanide® ISA 720
Source: PLoS One. 2011 Sep 19;6(9):e24413. doi: 10.1371/journal.pone.0024413 (PMC3176224; doi:10.1371/journal.pone.0024413)
Supplement: Protocol S3 — Revision two of the Protocol (DOC) [file pone.0024413.s004.doc]

A PHASE 1, RANDOMISED, DOUBLE BLINDED WITHIN DOSE, CONTROLLED, DOSE-ESCALATION, SAFETY AND IMMUNOGENICITY STUDY OF A BLOOD-STAGE VACCINE (MSP2-C1/ISA720) AGAINST *PLASMODIUM FALCIPARUM* MSP2 IN HEALTHY VOLUNTEERS

Version: 3.0

Date: 9 April, 2008

Q-Pharm Protocol Number: QP07C08

Proposed Start Date: October 2007

Proposed End Date: February 2009

Sponsored by:

Queensland Institute of Medical Research (QIMR), Australia

Funding Sponsor:

The PATH Malaria Vaccine Initiative (MVI)

**Confidentiality Statement**

This document is confidential and is to be distributed for review only to investigators, potential investigators, consultants, study staff, and applicable independent ethics committees or institutional review boards. The contents of this document shall not be disclosed to others without written authorization from QIMR (or others, as applicable), unless it is necessary to obtain informed consent from potential study participants.

**Team Roster**

**Principal Investigators:** Dr James McCarthy MBBS &

Dr Joanne Marjason, MBBS

Q-Pharm Pty Ltd

QIMR-Clive Berghofer Cancer Research Centre

Level D, 300 Herston Road

Herston, QLD, 4006

Australia

Phone: 041 442 4659

+61- (0)7 3845 3636 ( Q-Pharm)

E-mail: *J.McCarthy@uq.edu.au*

*j.marjason@qpharm.com.au*

**Clinical Investigators:** Dr Frances Jenkins, MBBS

Dr Richard Barr, MBBS

Q-Pharm Pty Ltd

QIMR-Clive Berghofer Cancer Research Centre

Level D, 300 Herston Road

Herston, QLD, 4006

Australia

**Local Medical Monitor:** Anthony Allworth, MBBS FRACP FRCPA

Royal Brisbane and Women’s Hospital

Bowen Bridge Road.

Herston QLD 4029 Australia

Phone: +61-(0)7 3636 8761

Fax: +61-(0) 7 3636 1388

E-mail: Tony_Allworth@health.qld.gov.au

**Statistician:** Mervyn R Thomas D. Phil

Emphron Informatics Pty Ltd

Level 3, 88 Jephson St

Toowong, QLD 4066

Australia

Ph: +61-(0)7 3331 3911

**Participating Site**

**Clinical Trial Site:** Q-Pharm Pty Ltd

QIMR-Clive Berghofer Cancer Research Centre

Level D, 300 Herston Rd

Herston, QLD, 4006

Australia

**Participating Institutional Review Boards**

**Ethical Review:** Queensland Institute of Medical Research-Human Research Ethics Committee (QIMR-HREC)

P.O. Royal Brisbane Hospital

Herston, QLD, 4029 Australia

Western Institutional Review Board® (WIRB)®

3535 Seventh Avenue SW

Olympia, Washington 98508-2029 USA

Walter and Eliza Hall Institute –

Human Research Ethics Committee

1G Royal Parade, Parkville

VIC, 3050Australia

**Participating Laboratories**

**Clinical Laboratory:** Queensland Health Pathology Service

Level 4, Block 7,

Royal Brisbane and Women's Hospital
 Herston Road,

Herston, QLD, 4029 Australia

**Immunology Laboratories:** Dr James Beeson

Walter and Eliza Hall Institute

Infection and Immunity Division

1G Royal Parade, Parkville

VIC, 3050 Australia

Dr Patrick Hogan

QHPS- Immunology Dept

Queensland Health Pathology Services

Bowen Bridge Road.

Herston, Brisbane QLD, 4006

Australia,

Dr Carole Long

Laboratory of Malaria and Vector Research (LMVR)

NIAID/NIH

12735 Twinbrook Parkway, Twinbrook III

Rockville, MD 20852 USA

**Sponsor Project Coordinators**

**CNS:**  Gabrielle McKee

Associate Director Clinical Operations

Clinical Network Services (CNS) Pty Ltd

Level 3, 88 Jephson Street

Toowong QLD, 4066 Australia

Phone: +61-(0)7 3331 3933

Mobile: +61-(0)400 915 551

Fax: +61-(0)7 3870 0520
 E-mail: *Gabrielle.McKee@clinical.net.au*

**MVI:**  Elissa Malkin, D.O., MPH

Senior Medical Officer

7500 Old Georgetown Road, 12^th^ Floor

Bethesda, MD 20814 USA

Phone: +1-240-395-2700

E-mail: [*emalkin@malariavaccine.org*](mailto:emalkin@malariavaccine.org)

Table of Contents

1.0 Objectives [2](#__RefHeading___Toc194823187)

1.1 Primary Objective [2](#__RefHeading___Toc194823188)

1.2 Secondary Objectives [2](#__RefHeading___Toc194823189)

1.2.1. Characterise the humoral and cellular immune responses to the
MSP2-C1/ISA720 vaccine [2](#__RefHeading___Toc194823190)

1.2.2. Determine the vaccine dose level that induces the highest serum
IgG concentration to MSP2 by ELISA [2](#__RefHeading___Toc194823191)

2.0 Introduction [2](#__RefHeading___Toc194823192)

2.1 Background [2](#__RefHeading___Toc194823193)

2.2 Vaccine Description [2](#__RefHeading___Toc194823194)

2.2.1 MSP2 Antigens [2](#__RefHeading___Toc194823195)

2.2.2 Adjuvant [2](#__RefHeading___Toc194823196)

2.2.3 Vaccine Formulation [2](#__RefHeading___Toc194823197)

2.3 Rationale [2](#__RefHeading___Toc194823198)

2.3.1 Preclinical Experience with MSP2-C1 [2](#__RefHeading___Toc194823199)

2.3.2 Clinical Experience with Other MSP2 Vaccines [2](#__RefHeading___Toc194823200)

2.3.3 Clinical Experience with Montanide® ISA720 [2](#__RefHeading___Toc194823201)

3.0 Study Design [2](#__RefHeading___Toc194823202)

3.1 Overall Design [2](#__RefHeading___Toc194823203)

3.2 Sample Size and Estimated Duration of Study [2](#__RefHeading___Toc194823204)

3.3 Group Allocation, Randomisation and Blinding [2](#__RefHeading___Toc194823205)

4.0 Selection and Enrolment of Volunteers [2](#__RefHeading___Toc194823206)

4.1 Inclusion Criteria [2](#__RefHeading___Toc194823207)

4.2 Exclusion Criteria [2](#__RefHeading___Toc194823208)

4.3 Treatments that could Potentially Interfere with Vaccine-Induced Immunity [2](#__RefHeading___Toc194823209)

4.4 Contraindications to Vaccination [2](#__RefHeading___Toc194823210)

4.5 Indications for Deferral of Vaccination [2](#__RefHeading___Toc194823211)

4.6 Subject Withdrawal Criteria [2](#__RefHeading___Toc194823212)

5.0 Vaccine Preparation [2](#__RefHeading___Toc194823213)

5.1 Supplies [2](#__RefHeading___Toc194823214)

5.2 Vaccine Storage [2](#__RefHeading___Toc194823215)

5.3 Vaccine Preparation [2](#__RefHeading___Toc194823216)

5.4 Vaccine Accountability [2](#__RefHeading___Toc194823217)

5.5 Disposition of Used/Unused Supplies [2](#__RefHeading___Toc194823218)

6.0 Study Procedures [2](#__RefHeading___Toc194823219)

6.1 Recruitment and Enrollment [2](#__RefHeading___Toc194823220)

6.2 Screening (Up to 28 Days Prior to Vaccination) [2](#__RefHeading___Toc194823221)

6.3 Immunisation Procedure [2](#__RefHeading___Toc194823222)

6.4 Clinical Monitoring and Evaluation [2](#__RefHeading___Toc194823223)

6.5 Volunteer Symptom Diary [2](#__RefHeading___Toc194823224)

6.6 Laboratory Testing [2](#__RefHeading___Toc194823225)

6.7 Immunologic Testing [2](#__RefHeading___Toc194823226)

6.7.1 Antibody Assays [2](#__RefHeading___Toc194823227)

6.7.1.1 Enzyme Immunoassays (ELISA) [2](#__RefHeading___Toc194823228)

6.7.1.2 Immunofluorescence microscopy [2](#__RefHeading___Toc194823229)

6.7.2 Western blotting of parasite protein extracts [2](#__RefHeading___Toc194823230)

6.7.3 Growth Inhibition Assay [2](#__RefHeading___Toc194823231)

6.7.4 Cell Mediated Immunity (CMI) [2](#__RefHeading___Toc194823232)

6.8 Use, Storage, and Tracking of Specimens and Data [2](#__RefHeading___Toc194823233)

7.0 Adverse Events Monitoring and Reporting [2](#__RefHeading___Toc194823234)

7.1 Definitions [2](#__RefHeading___Toc194823235)

7.1.1 Adverse Event (AE) [2](#__RefHeading___Toc194823236)

7.1.2 Serious Adverse Event (SAE) [2](#__RefHeading___Toc194823237)

7.2 Assessment of Adverse Events [2](#__RefHeading___Toc194823238)

7.2.1 Identification of AEs [2](#__RefHeading___Toc194823239)

7.2.2 Determination of Severity [2](#__RefHeading___Toc194823240)

7.2.3 Association with Receipt of the Study Vaccine [2](#__RefHeading___Toc194823241)

7.3 Adverse Event Reporting [2](#__RefHeading___Toc194823242)

7.4 Adverse Event Monitoring [2](#__RefHeading___Toc194823243)

7.4.1 Local Medical Monitor [2](#__RefHeading___Toc194823244)

7.4.2 Safety Monitoring Committee [2](#__RefHeading___Toc194823245)

7.5 Criteria for Placing the Study on Hold [2](#__RefHeading___Toc194823246)

7.5.1 Criteria for Stopping an Individual Volunteer’s Further
Vaccination [2](#__RefHeading___Toc194823247)

7.5.2 Process for Study Discontinuation [2](#__RefHeading___Toc194823248)

8.0 Data Collection and Monitoring [2](#__RefHeading___Toc194823249)

8.1 Source Documentation [2](#__RefHeading___Toc194823250)

8.2 Study Documentation [2](#__RefHeading___Toc194823251)

8.3 Access to Study Documentation: [2](#__RefHeading___Toc194823252)

8.4 Clinical Database [2](#__RefHeading___Toc194823253)

8.5 Retention of Records [2](#__RefHeading___Toc194823254)

8.6 Protocol Revisions [2](#__RefHeading___Toc194823255)

8.7 Clinical Investigator’s Brochure [2](#__RefHeading___Toc194823256)

8.8 Study Monitoring [2](#__RefHeading___Toc194823257)

9.0 Statistical Considerations [2](#__RefHeading___Toc194823258)

9.1 General Design [2](#__RefHeading___Toc194823259)

9.1.1 Description of the Statistical Methods to Be Employed [2](#__RefHeading___Toc194823260)

9.1.2 Analyses for Primary Objective (safety) [2](#__RefHeading___Toc194823261)

9.1.3 Analyses for Secondary Objectives (Immunogenicity) [2](#__RefHeading___Toc194823262)

9.1.3.1 Characterise the humoral and cellular immune responses to the
MSP2-C1/ISA720 vaccine: [2](#__RefHeading___Toc194823263)

9.1.4 Sample Size [2](#__RefHeading___Toc194823264)

10.0 Protection of Human Subjects [2](#__RefHeading___Toc194823265)

10.1 Institutional Review Board/Human Research Ethics Committee [2](#__RefHeading___Toc194823266)

10.2 Informed Consent [2](#__RefHeading___Toc194823267)

10.3 Risks [2](#__RefHeading___Toc194823268)

10.3.1 Venipuncture [2](#__RefHeading___Toc194823269)

10.3.2 Immunisation [2](#__RefHeading___Toc194823270)

10.3.3 Abnormal muscle enzyme levels [2](#__RefHeading___Toc194823271)

10.4 Precautions Taken to Minimize Risks [2](#__RefHeading___Toc194823272)

10.5 Benefits [2](#__RefHeading___Toc194823273)

10.6 Confidentiality [2](#__RefHeading___Toc194823274)

10.7 Compensation [2](#__RefHeading___Toc194823275)

10.8 Liability/Indemnity/Insurance [2](#__RefHeading___Toc194823276)

11.0 References [2](#__RefHeading___Toc194823277)

**TRIAL ASSURANCE: Investigator Agreement**

| **Title** | A PHASE 1 RANDOMISED, DOUBLE BLINDED WITHIN DOSE, CONTROLLED, DOSE-ESCALATION, SAFETY AND IMMUNOGENICITY STUDY OF A BLOOD-STAGE VACCINE (MSP2-C1/ISA720) AGAINST *PLASMODIUM FALCIPARUM* MSP2 IN HEALTHY VOLUNTEERS |
| --- | --- |

**I agree:**

1. To assume responsibility for the proper conduct of the study at this site.
2. To conduct the study in compliance with this protocol, any mutually agreed future protocol amendments, and with any other study conduct procedures provided by the sponsor.
3. Not to implement any changes to the protocol without agreement from the sponsors and prior review and written approval from the relevant study ethical review committees, except where necessary to eliminate an immediate hazard to the participants, or for administrative aspects of the study (where permitted by all applicable regulatory requirements).
4. To be thoroughly familiar with the appropriate use of the vaccine, as described in this protocol, and any other information provided by the sponsors, including, but not limited to,: the current Investigator’s Brochure (IB) and any IB supplement (if applicable).
5. To comply with, ICH and current “Good Clinical Practices” (GCP), the Declaration of Helsinki and the NHMRC National Statement on Ethical Conduct in Research Involving Humans and all applicable regulatory requirements.

| **Principal Investigators:** | Date: |  |
| --- | --- | --- |
| James McCarthy M.B.,B.S |  |  |
|  | Date: |  |
| Joanne K. Marjason M.B.,B.S |  |  |
|  |  |  |

Table of Abbreviations

AE Adverse Event

AST Aspartate aminotransferase

ALT Alanine aminotransferase

CMI Cell mediated immunity

CPK Creatinine Phosphokinase

CRF Case Report Form

ELISA Enzyme linked immunosorbent assay

GCP Good Clinical Practices

GMP Good Manufacturing Practices

GIA Growth Inhibition Assay

B-hCG Human choriogonadotropin

HBV Hepatitis B virus

HCV Hepatitis C virus

HIV Human immunodeficiency virus

HREC Human Research Ethics Committee (IRB)

ICH International Conference on Harmonization

IFA Immunofluorescence assay

IM Intramuscular

IRB Institutional Review Board (HREC)

Montanide® Seppic, France

MSP2 Merozoite Surface Protein 2

MSP2-C1/ISA720 Malaria Vaccine -equal protein concentrations of 2 allellic forms of MSP2: 3D7+FC27 in Montanide ISA720 adjuvant

MVI Malaria Vaccine Initiative

NHMRC National Health and Medical Research Council

NIH National Institutes of Health

PATH Program for Appropriate Technologies in Health

PI Principal Investigator

QIMR Queensland Institute of Medical Research

PBS Phosphate-buffered saline

SAE Serious Adverse Event

SMC Safety Monitoring Committee

SOP Standard Operating Procedure

QIMR Queensland Institute of Medical Research

PATH Program for Appropriate Technologies in Health

WEHI Walter and Eliza Hall Institute

WHO World Health Organization

WIRB Western Institutional Review Board

**Protocol Summary**

**Protocol Title:** A Phase 1 randomised, double blinded within dose, controlled, dose-escalation, safety and immunogenicity study of a blood-stage vaccine (MSP2-C1/ISA720) against *Plasmodium falciparum* MSP2 in healthy volunteers (QP07C08)

**Version Number and Date:** Version 3.0, 9 April 2008

**Volunteers:** Healthy malaria-naive male and non-pregnant female volunteers 18 to 45 years of age

**Number of Volunteers:** 45

**Trial Design:** Phase 1, Randomised Double blind Within Dose, Controlled, Dose Escalation Study in healthy volunteers

**Immunisation Schedule:**

| **Group** | **Number of Volunteers** | **Immunisation Schedule** | | | |
| --- | --- | --- | --- | --- | --- |
|  |  | **Day 0** | **12 Weeks** | | **24 Weeks** |
| 1 | 15 | A (12) + D (3) | | A (12) + D (3) | A (12) + D (3) |
| 2 | 15 | B (12) + D (3) | | B (12) + D (3) | B (12) + D (3) |
| 3 | 15 | C (12) + D (3) | | C (12) + D (3) | C (12) + D (3) |
| Total | 45 | A: 10 μg MSP2-C1/ ISA720  B: 40 μg MSP2-C1/ ISA720  C: 80 μg MSP2-C1/ ISA720  D: ISA720 Control | | | |

**Product Description:** The vaccine preparations to be studied contain an equal mixture of MSP2 from two different lines of *Plasmodium falciparum* (3D7 and FC27), both produced separately as recombinant proteins expressed by *E. coli*. Bulk MSP2 antigens were purified from lysates of transformed bacteria grown in a 50 L fermenter. The MSP2 protein was purified from this mixture by a combination of metal-chelate, anion-exchange and reverse-phase chromatography. Purified MSP2-3D7 and MSP2 FC27 were subsequently mixed and emulsified with Montanide® ISA720 (SEPPIC, France) as a final injectable formulation at 20 μg/ml, 80 μg/ml or 160 μg/ml. ISA720 Control vaccine consists of the buffer formulated in Montanide® ISA720 (SEPPIC) (30/70 w/w)

**Time Period:** A total of 65 weeks including screening vaccinations, study procedures, and follow-up of all volunteers. Each volunteer will be followed for a total of 48 weeks (maximum of 52 weeks including screening).

# Objectives

## 1.1 Primary Objective

To assess the safety and tolerability of 3 immunisations at 3 dose levels of the MSP2-C1/ISA720 vaccine in healthy adult malaria-naïve volunteers.

## 1.2 Secondary Objectives

### 1.2.1. Characterise the humoral and cellular immune responses to the MSP2-C1/ISA720 vaccine

### Determine the vaccine dose level that induces the highest serum IgG concentration to MSP2 by ELISA

Table 1: MSP2-C1/ISA720 Vaccine Dose Escalation Schedule

| Time  (Week) | Cohort 1: (n = 15) | Cohort 2: (n = 15) | Cohort 3: (n = 15) |
| --- | --- | --- | --- |
|  | 10 μg MSP2-C1/ISA720 | 40 μg MSP2-C1/ISA720 | 80 μg MSP2-C1/ISA720 |
| 0 | Vaccination 1 (n=15) |  |  |
|  | | | |
| 4 |  | Vaccination 1 (n=5) |  |
|  | | | |
| 8 |  | Vaccination 1 (n=10) |  |
| 11 | SMC | SMC |  |
| 12 | Vaccination 2 (n=15) |  |  |
| 13 |  |  | Vaccination 1 (n=5) |
|  | | | |
| 16 |  | Vaccination 2 (n=5) |  |
| 17 |  |  | Vaccination 1 (n=10) |
|  | | | |
| 20 |  | Vaccination 2 (n=10) |  |
| 24 | Vaccination 3 (n=15) | SMC | SMC |
| 25 |  |  | Vaccination 2 (n=5) |
|  | | | |
| 28 |  | Vaccination 3 (n=5) |  |
| 29 |  |  | Vaccination 2 (n=10) |
|  | | | |
| 32 |  | Vaccination 3 (n=10) |  |
|  | | | |
| 36 |  | SMC | SMC |
| 37 |  |  | Vaccination 3 (n=5) |
|  | | | |
| 41 |  |  | Vaccination 3 (n=10) |
|  | | | |
| 48 | Last Visit |  |  |
|  | | | |
| 52 |  | Last visit (n=5) |  |
|  | | | |
| 56 |  | Last visit (n=10) |  |
|  | | | |
| 61 |  |  | Last visit (n=5) |
|  | | | |
| 65 |  |  | Last visit (n=10) |

**** Table 1- Timing of vaccinations shown as a minimum. First Vaccinations may be delayed.**

# 2.0 Introduction

## 2.1 Background

The World Health Organisation reported in 2005 that malaria kills more than 1 million people annually and that approximately 3.2 million people living in 107 countries or territories are at risk of infection [1]. Most of the malaria mortality occurs in sub‑Saharan Africa and in children under 5 years of age. Of the four species of malaria parasite that infect humans, *Plasmodium falciparum* is responsible for the majority of these deaths. Mounting drug resistance of the malaria parasite, as well as widespread resistance of mosquitoes to insecticides, make these control strategies increasingly unrealistic. A vaccine that would reduce both mortality and morbidity secondary to *P. falciparum* infection would be a valuable resource in the fight against this disease.

*P. falciparum* has a complex life cycle. Sporozoites, the infectious stage of the parasite, are transmitted to humans through the saliva of infected female mosquitoes while taking a blood meal. Sporozoites travel through the bloodstream to the liver, where they invade hepatocytes and then multiply asexually into merozoites. Six to 10 days after invasion, the hepatocytes rupture, releasing thousands of merozoites into the bloodstream. These merozoites invade erythrocytes, multiply and after 2 days, release progeny merozoites, which subsequently invade new erythrocytes to continue the asexual blood-stage cycle. Clinical symptoms in humans are due to this asexual blood-stage of the parasite’s life cycle. A small percentage of merozoites do not multiply after invading erythrocytes, but instead differentiate into gametocytes. These gametocytes are ingested by a female mosquito during a subsequent blood meal and undergo sexual reproduction in the mosquito midgut, producing a zygote. The zygote matures and releases sporozoites that migrate to the mosquito’s salivary glands, thus completing the life cycle.

The complex life-cycle of the malaria parasite offers several points that could be targeted by vaccine-induced immune responses but the merozoite, which invades the host’s erythrocytes, is considered a particularly important target. MSP2 (Merozoite Surface Protein 2), the antigen in the vaccine to be tested in this study, is one of several proteins on the merozoite surface that are being assessed as components of a vaccine against malaria. MSP2 is a ~28kDa protein which is anchored in the membrane of the merozoite by a C-terminal glycosylphosphatidyl-inositol moiety (2, 3). MSP2 has been detected in all isolates of *P. falciparum* examined and appears essential for parasite viability as attempts to "knock-out" the gene have not been successful (4). The function of MSP2 is unknown but it appears to be specific for *P. falciparum* as orthologues of MSP2 have not been detected in *P. vivax*, or rodent and simian parasites, with the exception of *P. reichenowi* a malaria parasite infecting chimpanzees (5).

MSP2 is highly polymorphic with a central variable region flanked by conserved N- and C-terminal regions. The extremely large number of MSP2 alleles fall into two allelic families, 3D7 and FC27, characterized by different centrally-located sequence repeats and family-specific non-repetitive sequences flanking the repeats (6, 7) and the current vaccine contains a representative of these two families of MSP2 alleles.

Being an abundant protein on the merozoite surface MSP2 is a potential target of antibodies induced by infection or vaccination. The majority of individuals living in regions where there is intense transmission of *P. falciparum* develop high titres of anti-MSP2 antibodies in response to repeated infections by five years of age. Much of the natural anti-MSP2 antibody response is directed against epitopes in the central variable region of the molecule (8-10). Several sero-epidemiological studies have shown an association between antibody responses to MSP2 and resistance to infection or disease (e.g. 11-13). The importance of MSP2 as a probable target of naturally acquired protective immunity is also supported by the patterns of various MSP2 genotypes seen among infected individuals (14). Although some anti-MSP2 monoclonal and polyclonal antibodies have been reported to inhibit merozoite invasion *in vitro* (15-17) we have not been able to demonstrate inhibition of invasion with antibodies raised in rabbits to recombinant forms of MSP2 including the 3D7 and FC27 forms of MSP2 to be tested in this vaccine. This may be because only those anti-MSP2 antibodies with a particular fine specificity are effective at blocking merozoite invasion or because other effector mechanisms, such as antibody dependent cellular inhibition are operating (18)

## 2.2 Vaccine Description

### 2.2.1 MSP2 Antigens

Both EcMSP2-3D7 and EcMSP2-FC27 are highly purified ~24 kDa proteins that correspond to the mature polypeptides of *P. falciparum* (3D7) MSP2 and *P. falciparum* (FC27) MSP2, respectively. Each protein has added a 6-histidine C-terminal tag to allow purification by metal-chelate chromatography. EcMSP2-3D7 has a molecular mass of 24,189 Da, whereas EcMSP2-FC27 has a molecular mass of 23,843 Da. The full details of the sequences of the two antigens are described in the Investigational Brochure.

The two forms of MSP2 were separately expressed in *E. coli* from synthetic genes cloned into the *E. coli* expression plasmid pET22b. The two recombinant proteins were purified from *E. coli* lysates by a combination of chelating, anion-exchange and reverse-phase chromatography. The purification processes were designed to separate monomeric MSP2 from non-product related components (e.g. *E. coli* proteins, DNA and endotoxin), as well as MSP2 aggregates or fragments.

Both recombinant forms of MSP2 have a propensity to form fibrils, which have some but not all of the characteristics of amyloid fibrils. A small amount of the formulated antigen may be in this fibrillar form but independent experts do not consider that this poses significant safety concerns and it is unlikely to invalidate the assessment of immunogenicity.

### 2.2.2 Adjuvant

Montanide® ISA720 (ISA720) is an experimental adjuvant used for generating water-in-oil emulsions. ISA720 contains the metabolizable oil squalene and a surfactant from the mannide monooleate family. Homogenization of the antigens in aqueous buffer with Montanide® ISA720 was used to generate an emulsion with a droplet size of approximately 1 micron, which the manufacturer, SEPPIC Inc., recommends as optimal for stability and immunogenicity. Glycine (final concentration 5 mM) was added to the aqueous phase prior to homogenization because this has been shown to prevent antigen modifications that can occur in Montanide® ISA720 emulsions (19). Antigens in ISA720 emulsions have been shown to induce high antibody titers in several animal species. The formation of a depot at the injection site is considered important for adjuvant action. In addition, the stimulation of antigen presenting cells and other mechanisms may also play a role in adjuvant activity of ISA720 formulations. Vaccine formulations using ISA720 as an adjuvant have been given safely to humans, and have been used in human trials with a variety of experimental malaria vaccines, including a combination vaccine consisting of MSP1, MSP2 and RESA (20-32), as well as with a candidate HIV vaccine (33).

### 2.2.3 Vaccine Formulation

For the formulation of the vaccine, EcMSP2-3D7 and EcMSP2-FC27 were mixed in equal amounts and emulsified with the Montanide ISA720 to generate the final drug product. As the trial is a dose-escalating study three different formulations were prepared, with total protein concentrations (EcMSP2-3D7 plus EcMSP2-FC27) of 10 μg, 40 μg and 80 μg per 0.5 ml dose. For the ISA720 Control, the aqueous buffer without antigen was emulsified with ISA720. The formulated vaccines were dispensed without preservative into single dose vials and stored at 2-8^o^C.

## 2.3 Rationale

### 2.3.1 Preclinical Experience with MSP2-C1

The EcMSP2-3D7 and EcMSP2-FC27 antigens have been tested individually or in combination using various adjuvants in rabbits, mice and guinea pigs. The combination of the two antigens (MSP2-C1) formulated in ISA720 has been tested in rabbits and mice. A total of six individual preclinical animal studies have been conducted to assess the safety, immunogenicity or toxicity of the MSP2-C1 or the individual antigens formulated in ISA720. These studies have demonstrated the safety of MSP2-C1 when adjuvanted with Montanide® ISA720. Immunogenicity data from rabbits and mice have demonstrated that each animal species developed anti-MSP2 antibody responses without the occurrence of clinically significant safety issues. Repeated intramuscular administration of the maximum human dose to rabbits in a toxicology study did not result in any specific signs of systemic or local toxicity.

### 2.3.2 Clinical Experience with Other MSP2 Vaccines

The MSP2-C1 combination vaccine formulation that will be used in this study has not yet been tested in human trials. However, both Phase 1 and Phase 2 studies have been carried out previously with vaccines containing a recombinant form of 3D7 MSP2 known as Ag1624. Ag1624, like the forms of MSP2 in the current vaccine was expressed in *E. coli* and was purified by a series of chromatography steps. Ag1642 differs from EcMSP2-3D7 and ECMSP2-FC27 in that the hexa his tag is located at the N-terminus rather than the C-terminus. Also, a small number of additional residues encoded by the plasmid cloning vector are present at both the N- and C-terminus in Ag1624.

Recombinant MSP2 was injected into humans for the first time when Ag1624 in combination with recombinant CS protein (Combination A), formulated with alum was tested in healthy Swiss volunteers (20). Thirty three participants received two low (20 μg) or two high (100 μg) vaccinations intramuscularly, while six volunteers received placebo (alum alone). Twenty-six participants reported 51 immunisation-related adverse events, mainly pain at the injection site. There were no serious adverse events and the vaccine was considered safe for further evaluation. However, the development of this vaccine ceased because immune responses were poor and no protection was observed among five subjects who were experimentally challenged with *P. falciparum*.

Subsequently, a series of Phase 1 and 2 trials were carried out with a combination of three recombinant antigens (Combination B) that included 3D7 MSP2. In addition to Ag1624 Combination B contained 190L (a small fragment of MSP1) and Ag1505 (a large fragment of RESA). All three components of Combination B were formulated as a water-in-oil emulsion with Montanide® ISA720, the adjuvant to be used in this study. Two Phase I vaccine trials were conducted at the Royal Brisbane Hospital to test the immunogenicity and safety of the Combination B vaccine in healthy Australian volunteers (21).

The first trial tested the safety and immunogenicity of the mixture of three antigens given at a single site or given separately at three sites. For the first vaccination, volunteers received 3 x 100 μg of antigen or 3 x 50 μg; all received 3 x 20 μg in the second vaccination. In the second trial, which compared the safety and immunogenicity of different doses of a mixture of the three antigens, the subjects received 3 x 50 μg, 3 x 13 μg or 3 x 4 μg for the first vaccination and 3 x 20 μg, 3 x 13 μg or 3 x 4 μg for the second vaccination, respectively. No serious adverse events were reported in either trial but local reactions, particularly pain and tenderness, occurred in the majority of subjects, These reactions lasted for up to a few days and were also seen in subjects receiving the adjuvant alone. One subject had a local reaction that was severe for three days before resolving gradually over three weeks.

In both trials some subjects had delayed local reactions with pain, tenderness and/or swelling developing 10 days or later after vaccination. These reactions were clearly related to antigen dose with the majority occurring in subjects receiving the mixture of 3 x 100 μg antigen in a single site and none occurring in subjects receiving the lowest antigen dose or the adjuvant alone. The delayed reactions were mild to moderate in all but two subjects. One developed severe pain and swelling in the injected thigh 10 days after the vaccination, which resolved over a week. This subject was given 20 μg of each antigen as a second vaccination and six weeks later developed a hard painful mass at the second injection site. The pain and the mass resolved over a few weeks with no biochemical evidence of muscle damage. The other with a severe delayed reaction developed pain and swelling in the thigh and around the knee two weeks after vaccination. There was marked discomfort on use. Symptoms resolved over two weeks with no biochemical evidence of muscle damage.

In a subsequent placebo controlled, double blind trial with Combination B at the Royal Brisbane Hospital, the vaccine was tested for its ability to inhibit parasite growth rates in human volunteers (22). Volunteers each received two vaccinations, 6 weeks apart with 13 µg of each component of Combination B or adjuvant alone and four weeks later were challenged intravenously with approximately 100 *P. falciparum*-infected red cells. Parasitaemias were monitored with a quantitative PCR assay (23) from day four until day eight when the volunteers were treated to eliminate the infection. No significant decrease in parasite growth rates was seen, nor was there any significant correlation between growth rates and any of the measured immunological responses. These results suggested that formulations with significantly greater immunogenicity would be required for these antigens to provide a protective effect. Of the 12 volunteers in this challenge trial the majority had localized pain and tenderness after vaccination which in one was sufficient to preclude a second injection. No other adverse events judged to be related to the vaccine were observed. A second volunteer had a moderate local reaction after the second injection which resolved over seven days.

Although the Combination B vaccine failed to reduce parasite growth rates in Australian volunteers it was considered possible that the vaccine may be more effective in individuals living in a malaria-endemic setting. For this reason Combination B, again with Montanide® ISA720 as the adjuvant, was assessed in two trials in Papua New Guinea. The first of these trials (24) was a Phase 1b study in which 10 adult males from the Wosera region of the East Sepik province were injected twice, four weeks apart, with the vaccine; two individuals were injected with the adjuvant alone. While good cellular immune responses were seen to RESA and MSP1 the response to MSP2 was poor, with no change observed in the anti-MSP2 antibody titres compared to baseline values. Although no systemic reactions occurred, approximately half the subjects had mild pain at the injection site in the few days following vaccination.

Subsequently, a phase 1-2b double-blind randomised placebo-controlled trial was carried out in 120 5-9 year-old children living in the Wosera (25). The subjects receiving the vaccine (n=60) were injected intramuscularly with 15 μg of each antigen on two occasions four weeks apart. The same number of children received the placebo, physiological saline emulsified with Montanide ® ISA720. The vaccine induced significant antibody responses to all three antigens; no severe or serious adverse events occurred in children who received vaccine or placebo (26). In this trial there was a 62% reduction in parasite density among children who were not pretreated with sulfadoxine-pyrimethamine. The vaccinated children had a lower prevalence of parasites carrying the 3D7 MSP2 allelic form (corresponding to that in the vaccine) and a higher incidence of symptomatic malaria associated with parasites of the FC27 MSP2 allelic form. This result is the primary rationale for proceeding with the development of the MSP2-C1 vaccine to be tested in the current study.

### 2.3.3 Clinical Experience with Montanide® ISA720

In addition to the Combination B trials discussed above Montanide® ISA720 formulations of five other experimental malaria vaccines (27-32) and one experimental HIV vaccine (33) have been tested in humans. The antigens in these vaccines have been either recombinant proteins or long synthetic peptides. Including the Combination B trials discussed above, Montanide® ISA720, with or without antigens has been given to more than 450 subjects, 120 of whom were children aged 5-9 in the Combination B Phase 1-2b trial in Papua New Guinea. In these trials more than 200 volunteers have received Montanide® ISA720 without any antigen, the largest volume being 1.8 ml. The adjuvant alone was well tolerated with no SAEs reported. Antigens formulated in Montanide® ISA720 were also mostly well tolerated and no SAEs were reported. Systemic reactions such as fatigue, myalgia and headache occurred in a minority of volunteers and were not more common than for some commonly used vaccines.

The most common adverse event observed in trials using Montanide ISA720 is local reactogenicity (34). The acute reactions are characterized by of pain, swelling, induration, sterile abscess, and granuloma formation. These have been generally mild to moderate in 33 to 91% of volunteers. In the single Phase 2 trial in children in an endemic area the rate was 12% (26). The overall rate for mild and moderate acute local reactions is similar to that observed with licensed pediatric vaccines such as DT (standard pediatric dose) (55% mild/moderate local reactions) or dT (low dose vaccine) (52% mild/moderate reactions) in 6-year-old children. Severe local reactions occurred only with 5 vaccinees who received the vaccine via the intramuscular route for a cumulative rate of 1%. This proportion is not greater than that observed for some licensed vaccines (17% severe local reactions with DT and 11% severe local reactions with dT) (35). Severe local reactions were seen much more commonly in trials administering the vaccine subcutaneously. In these 2 trials (30) up to 44% of recipients of higher doses were not revaccinated due to local reactions. Acute local reactions were seen frequently with pain, swelling and/or induration most common. In the HIV vaccine study (33) sterile abscess and granuloma formation was seen in some volunteers, particularly those receiving the high antigen dose (2 mg), which was far in excess of that given in the malaria vaccine studies or that planned in this study. In most studies the vaccine was injected intramuscularly and when this route was used the majority of local reactions were mild to moderate with a cumulative rate of 1% for severe reactions. In contrast, severe local reactions were seen more commonly in the two studies in which the vaccine was administered subcutaneously (31, 32).

Delayed local reactions as seen in the Combination B studies (see Section 2.3.2) occurred in some volunteers in the majority of other trials with antigens formulated in Montanide® ISA720. Over all of the studies approximately 11% of volunteers developed these reactions, which were characterized by pain and swelling at the injection site occurring 10-28 days after vaccination. They resolved without sequelae usually in a few days but in some cases several weeks. There was a trend indicating that the rate and severity of these delayed reactions was increased with higher antigen doses and multiple injections.

One trial which assessed the safety and immunogenicity of AMA1 formulated in Montanide ISA720 was carried out at the Royal Brisbane Hospital (28). In this dose-escalating study three groups of volunteers were vaccinated intramuscularly with 5 μg (n=10), 20 μg (n=10) or 80 μg (n=9) of AMA1, respectively, in 0.5 ml of formulated vaccine at 0, 3 and 6 months. No vaccine-related adverse SAEs were recorded but most subjects had a mild to moderate, transient local reaction after the first vaccination. One subject with a severe local reaction after the first vaccination (5 μg) lasting five days was treated with acetylsalicylic acid and given no further doses of vaccine. Another subject developed a delayed local reaction 12 days after the third dose (80 μg) of vaccine. Discomfort persisted for two weeks, whereas swelling took more than 1 month to resolve.

# 3.0 Study Design

## 3.1 Overall Design

The study is a Phase 1 dose-escalating clinical trial in healthy adult malaria-naïve volunteers designed to evaluate the safety and immunogenicity of the MSP2-C1/ ISA720 malaria vaccine.

Volunteers will be recruited, and after providing written informed consent, will undergo eligibility screening, including medical history, physical examination, laboratory investigations including haematology testing, liver and renal function tests, pregnancy (females only), HIV, Hepatitis B and C screening and urinalysis. Those determined to be eligible, based on the inclusion and exclusion criteria described in **Section 4** in this protocol will be enrolled and randomised into the study. Participants will receive one of three dose regimens of the MSP2-C1/ISA720 vaccine or ISA720 as a control. Serum pregnancy tests will be performed on female volunteers of child bearing potential prior to enrollment and urine pregnancy testing prior to each vaccination; these females will be counselled to avoid becoming pregnant during the study. Clinically significant abnormalities will be reviewed by the investigators and referral for follow-up care will be provided as indicated. For eligible participants, the Day 0 visit will be scheduled for receipt of the first vaccination. Vaccinated volunteers will be observed for immediate reactions following each vaccination for 30 minutes. Volunteers will return to the clinic on Days 3, 7, 14 and 28 following each vaccination for clinical assessment, and will be given diary cards to record and grade local and systemic signs and symptoms. Phone contact will be made on D1 & D21 following each vaccination. Volunteers will also return 56 days following vaccinations 1 and 2 for screening prior to each subsequent vaccination. See **Appendix 1 Schedule of Assessments, and Table 1** for a tabular description of the vaccination schedule.

Forty-five volunteers will be enrolled and assigned to one of three dose groups (10, 40 or 80µg) or ISA720 Control as outlined in **Table 1**. All vaccines and the ISA720 Control will contain the same amount of Montanide® ISA720. As with other recombinant protein vaccines, hypersensitivity reactions would be expected to occur within the first 24 hours after receipt of the vaccine, and other severe local or systemic reactions within 72 hours. As outlined above, delayed local reactions have been observed in previous ISA720 vaccine studies. Thus, volunteers will be reviewed on days 3, 7, 14 and 28 following each vaccination, and vaccinations will be staggered within the two higher dose cohorts such that 4 volunteers will be vaccinated 4 weeks prior to the remaining 8 volunteers. Stopping rules as detailed in **Section 7.5** below will be in place to enable suspending the trial until review by the safety monitoring committee (SMC).

## 3.2 Sample Size and Estimated Duration of Study

A total of 45 volunteers will be vaccinated in 3 cohorts of 15 (see **Section 9.1.4** for a justification of the sample size). Twelve in each cohort will be randomized to receive 10, 40, or 80 µg of MSP2-C1 formulated in ISA720; 3 in each cohort will be randomized to receive ISA720 Control. The trial is expected to last for a total of 65 weeks. Each volunteer will be followed for 48 weeks from the time of the first injection.

It is estimated that up to 150 volunteers will need to be screened to complete enrolment.

## 3.3 Group Allocation, Randomisation and Blinding

The three dose groups will be screened and enrolled consecutively. Once screening has started and the first 15 participants have been deemed eligible, they will be assigned to Cohort 1 and a Day 0 visit will be scheduled. Alternates may also be identified and held in reserve. The first dose group will be vaccinated over the course of 1-2 days, with 2 participants completed a day prior to the remaining 13. If the alternates are not vaccinated, they will be invited to participate as members of the second or third cohort. The second and third dose cohorts will each be split into 2 subgroups for vaccination. The first 5 participants of both the 40 and 80 µg dose cohorts will be vaccinated at least 4 weeks after the last vaccination of the previous dose cohort and the remaining 10 participants in each dose cohort will be vaccinated at least 4 weeks after the first 4 participants in their cohort to maximize safety. After the first vaccination for each dose cohort, no replacement volunteers will be added if a subject withdrawals from the study.

In this trial, everyone will be blinded to the randomisation scheme except for the study pharmacist (or designee) who prepares the vaccine dose for administration. Randomization and blinding occur within each dose cohort. The study pharmacist/nominee will prepare identical syringes containing the study vaccines, according to a randomisation schedule provided by the study statistician, so that personnel who perform the study assessments do not know the identity of the study vaccine. Each participant considered eligible for vaccination will be assigned a randomisation number at Visit 1 (Day 0) (Section 6.4). The randomization number will used to identify the participant throughout the remainder of the study.

The volume of vaccine and ISA720 Control will be identical for each group. The study pharmacist will provide the person administering vaccine with the subject’s randomisation number, initials, and a blinded syringe containing the study medication. The pharmacist (or designee) will not inform any of the investigational staff of the treatment assignment and will not perform any other subject-related duties.

The Sponsor will provide the Investigator with code-break envelopes for individual participants. These should be retained intact and returned to the Sponsor with the completed Case Report Forms (CRFs) at the end of the study, unless the Investigator needs to break the code for an individual subject. Code-break envelopes for all participants will also be held by the sponsor and may be broken for an individual subject(s) or for the entire study at the request of the SMC to evaluate serious adverse events. The code for a subject should only be broken in exceptional circumstances, e.g. in the case of a medical emergency or where the Investigator feels that knowledge of the subject’s treatment will aid his/her management of the event. In the latter case, approval of the sponsor should be obtained whenever possible. Any code break should be documented in the study file, including a rationale for the break.

Allocation of the study subjects will not be disclosed to the scientists undertaking the exploratory immunological studies (anti-MSP2 antibody by ELISA, and CMI responses) until these test results are completed for each cohort. The GIA and IFA require unblinding and this can be done following the anti-MSP2 antibody assay completion.

# 4.0 Selection and Enrolment of Volunteers

Volunteers will be recruited from the database of healthy volunteers maintained by Q-Pharm, or by advertisement to students of The University of Queensland or to the general community, as approved by the Queensland Institute of Medical Research Human Research Ethics Committee (QIMR-HREC) and other respective IRBs reviewing the protocol.

## 4.1 Inclusion Criteria

1. Males or females between 18 and 45 years, inclusive.
2. Participants must be in good health, as assessed during pre-study medical examination and by review of screening laboratory tests.
3. Available for the duration of the trial (maximum of 52 weeks).
4. Participants must understand the procedures involved and agree to participate in the study by giving fully informed, written consent.
5. Agree to not donate blood during the course of the trial
6. Be contactable by telephone throughout the study

## 4.2 Exclusion Criteria

Participants may be excluded from the study either during screening, on vaccination days, or during the blood sampling intervals, for any of the following reasons:

1. History of malaria or malaria exposure within last two years
2. Travelled to or lived (>2 weeks) in a malaria-endemic country during the past 12 months or planned travel to a malaria-endemic country during the course of the study.
3. Significant intercurrent disease of any type, in particular liver, renal, cardiac, pulmonary, neurologic, rheumatologic, or autoimmune disease by history, physical examination, and/or laboratory studies including urinalysis.
4. Evidence of acute illness within the four weeks before trial prior to screening.
5. Use of corticosteroids, anti-inflammatory drugs, any immunomodulators or anticoagulants. Currently receiving or have previously received immunosuppressive therapy, including systemic steroids including ACTH or inhaled steroids in dosages which are associated with hypothalamic-pituitary-adrenal axis suppression such as 1mg/kg/day of prednisone or its equivalent or chronic use of inhaled high potency corticosteroids (budesonide 800 µg per day or fluticasone 750 µg).
6. Medical requirement for intravenous immunoglobulin or blood transfusions
7. History of a severe allergic reaction or anaphylaxis or convulsions following any vaccination.
8. Receipt of a live vaccine within past 4 weeks or a killed vaccine within past 2 weeks prior to entry into the study.
9. Participation in any clinical trial within the 4 weeks preceding the study.
10. Alcohol consumption greater than community norms (i.e. more than 21 standard drinks per week for males, or more than 14 standard drinks per week for females).
11. A history of drug habituation, or any prior intravenous usage of an illicit substance.
12. Smokers (>20 cigarettes/day) or those who are unable to refrain from smoking during the periods of confinement in each period of the study.
13. Positive test for HIV, hepatitis B, hepatitis C, or any clinically significant biochemical or haematological abnormality or any clinically significant abnormality in urinalysis.
14. Participant unwilling to regulate vigorous exercise within the vaccination period.
15. Female participant unwilling to use reliable contraception methods for the duration of the trial.
16. Pregnancy confirmed by a positive serum or urine human chorionic gonadotropin (ß-HCG), or breast-feeding or lactating females
17. Blood donation during the 8 weeks preceding the study.
18. Receipt on any investigational product four weeks prior to screening.
19. History of a known allergy to nickel.

## 4.3 Treatments that could Potentially Interfere with Vaccine-Induced Immunity

Participants’ use of concomitant medications will be checked at each visit. If any of the following criteria become applicable during the study, the participant will be excluded from receiving further doses of the study vaccine and will not be included in the immunogenicity evaluations after the time of exclusion. The participant will, however, be encouraged to remain in the safety evaluation for doses already received.

1. Use of any investigational drug or investigational vaccine other than the study vaccine during the study period.
2. Administration of a licensed vaccine during the period starting from 14 days before to 30 days after each vaccination
3. Administration of chronic (defined as more than 14 days) immunosuppressants or other immune-modifying drugs. (For corticosteroids, this will mean prednisone, or equivalent, greater than or equal to 10 mg/day. Topical and nasal steroids are allowed subject to dose limitations outlined in 4.2 Exclusion Criteria (v).)
4. Administration of immunoglobulins and/or any blood products until the last study visit.

## 4.4 Contraindications to Vaccination

The following criteria should be checked prior to each immunisation and are contraindications to further immunisation. However, the participant will be encouraged to remain in the safety evaluation for doses already received.

1. Hypersensitivity reaction following administration of the study vaccine.
2. Pregnancy, as determined by a positive urine β- (hCG).
3. Occurrence of severe chronic disease (such as diabetes or tuberculosis), which in the view of the investigators could jeopardize the safety of study participant or interpretation of the safety or immunogenicity data.

## 4.5 Indications for Deferral of Vaccination

The adverse events (AEs) outlined below constitute grounds for deferral of vaccine administration at that point in time. If any one of these AEs occurs at the time scheduled for vaccination, the participant may be vaccinated at a later date, within the allowable time interval specified in **Section 6.4** of this protocol, or withdrawn at the discretion of the investigator. The participant must be followed until resolution of the event as with any AE. If the participant is withdrawn from the study and has received at least one vaccine dose, he/she will be encouraged to remain in the safety evaluation for the duration of the study.

1. Oral temperature >37.5 °C will warrant deferral of immunisation until fever and causative illness resolve, in the opinion of the investigator.
2. Any other acute condition that in the opinion of the investigator poses a threat to the individual if immunized, or that may complicate interpretation of the safety of the vaccine following immunisation.
3. Such individual(s) will be followed by the investigators until the symptoms resolve or the window for immunisation expires. No further vaccination will be performed if the participant does not recover (oral temperature ≤37.5 °C and/or lack of symptoms) within the originally scheduled vaccination time interval. If the subject has received at least one dose of vaccine, he/she will be followed for evaluation of safety and immunogenicity. If the individual meets any of the above criteria for deferral on the day of first immunisation, as an alternative to deferral of vaccination, the investigator may instead elect to exclude the volunteer from further participation in the study. Eligible alternate(s) will then be vaccinated instead.

## 4.6 Subject Withdrawal Criteria

A volunteer will not be considered to have completed the trial if any of the circumstances outlined below apply. However, any volunteer who has received at least one dose of vaccine will be encouraged to remain in the safety evaluation for the duration of the study.

1. *Research terminated by sponsor, investigator, or regulatory body* – applies if the study is terminated by a sponsor, investigator, or regulatory body for any reason.
2. *Withdrawal of consent* – applies to a participant who withdraws consent to participate in the study for any reason.
3. *Noncompliant with protocol* – applies to a participant who does not comply with protocol-specific visits or evaluations, on a consistent basis, such that adequate follow-up is not possible and the participant’s safety would be compromised by continuing in the trial.
4. *Significant adverse event* - applies to a participant who is withdrawn from the study by the investigator due to an adverse event, serious or otherwise.
5. *Lost to follow-up* - applies to a participant who consistently does not return for protocol study visits, or who cannot be contacted or located.
6. If *new safety data* becomes available during the trial that impacts participant health.
7. *Pregnancy*. Should a female volunteer become pregnant during the course of the study, she will be withdrawn from the study and followed for the duration of the pregnancy. The outcome of the pregnancy will be documented.
8. If, in the judgment of the investigator, the subject has a condition that would interfere with safety or compliance with follow-up visits.
9. *Other* – is a category used when previous categories do not apply, and requires an explanation.

# 5.0 Vaccine Preparation

## 5.1 Supplies

Vaccine and ISA720 Control for this protocol will be supplied to the study site pharmacist by CSL Limited, where the vaccines were formulated, dispensed and labeled. Clinical Trial Material will be transported at 2^o^- 8^o^C from CSL Limited to the study site pharmacy under temperature controlled conditions and will be monitored with a temperature recording device. Upon receipt of Clinical Trial Material shipment, the study site pharmacist will check the shipment contents, complete the requirements for reporting shipment temperature monitoring and sign the enclosed Clinical Trial Material Dispatch Form. By signing this form, assurance will be provided that the vaccine supplies will be handled and stored safely and appropriately; were received undamaged and will only be dispensed to study patients in accordance with the protocol. The study site pharmacy will be responsible for maintaining the appropriate supply of vaccine at the site and will request any necessary additional Clinical Trial Material in writing through the sponsor.

To ensure that the temperature was maintained between 2^o^- 8^o^C during transit, the temperature recording device must be immediately returned to CSL Limited, whereby the temperature data will be downloaded and inspected. Written confirmation from CSL Limited will be supplied to the study site regarding the suitability of use for the Clinical Trial Material.

The MSP2-C1/ ISA720 malaria vaccine is supplied as a milky white emulsion in single‑dose vials. Each 5 mL vial contains 0.8 mL, of which 0.5 mL is the intended volume to be injected. 0.5 mL of vaccine contains the equivalent of 0.15 ml of buffer containing antigen emulsified in Montanide® ISA720. The MSP2-C1/ ISA720 vaccine and ISA720 Control conforms to established requirements for sterility, safety and identity.

## 5.2 Vaccine Storage

MSP2-C1/ ISA720 vaccine and ISA720 control should be maintained at 2 - 8^o^C until just prior to administration. Vaccine/ Control should NOT be frozen at any time. Single-dose vials should be stored in the upright position.

## 5.3 Vaccine Preparation

Vaccine/ Control will be prepared by the site pharmacist/ nominee for each participant according to the Master Randomisation Schedule. The Vaccine/ Control vial should be maintained at 2-8ºC until just prior to vaccination. The required vial will be removed from the refrigerated storage, gently inverted several times to warm the contents and then drawn up into a syringe. The site pharmacist/ nominee will apply a Participant specific label to the syringe confirming the Protocol Number, Participant Identification Number, Initials and vaccination date.

## 5.4 Vaccine Accountability

The site pharmacist/ nominee is responsible for maintaining an accurate inventory and accountability record of vaccine/ control supplies for this study. Partially used vials may not be administered to other volunteers.

## 5.5 Disposition of Used/Unused Supplies

After administration of vaccine/ control dose, the used vials will be stored in the study pharmacy at the study site, and will be accounted for and stored until monitoring by the study sponsor. Used vials may be disposed of according to study site protocol after monitoring has been completed. Final disposition of unused vaccine supplies will be determined by the Sponsor in conjunction with the trial site pharmacy.

# 6.0 Study Procedures

The following sections provide a detailed listing of the procedures and studies to be performed in this protocol at designated time points. Each volunteer will receive 3 immunisations at 12 week intervals and will be asked to return to the clinic for 16 visits. The total volume of blood is approximately **450** mL to be drawn over the duration of the trial (approximately 1 year). This is approximately the same volume collected when donating a single unit of blood and should not compromise the health of trial participants over this extended period.

## 6.1 Recruitment and Enrollment

Volunteers will respond to HREC-IRB approved advertisements distributed by the clinical trial site in local newspapers and posted flyers. A screening visit will be scheduled after an initial contact screen by clinical trial staff consisting of background information of the trial. During this initial screening visit, the volunteer will read the consent form and be encouraged to ask questions. Volunteer willing to be considered for participation may sign the screening consent form during the screening visit, or return after further consideration. The volunteer will be given a copy of the consent for their records. Rolling recruitment and enrollment will occur to fill the first dose cohort prior to the second dose cohort followed by the third dose cohort. After enrollment into a dose group, volunteers will be randomly assigned to receive either the MSP2-C1/ ISA720 vaccine or ISA720 Control.

On any given day of first vaccination, more volunteers (up to three) may be invited to participate than are scheduled to be vaccinated that day. If not vaccinated, these alternates will be invited to return on the next day of first vaccination. Alternates will be compensated for the study visit even if not vaccinated, as described in the participation information and consent form and Section 10.7.

## 6.2 Screening (Up to 28 Days Prior to Vaccination)

1. Explain the study via the Participation Informed Consent (Appendix 6) and gain Screening Informed Consent (Appendix 5) from the volunteer.
2. Ensure the subject has signed the Screening Informed Consent and received a signed copy.
3. Elicit a complete medical history, including menstrual and contraceptive history and/or history of surgical sterility for female participants. Counsel females of childbearing potential to utilize effective contraception during the study.
4. Elicit a social history including smoking and alcohol use
5. Undertake a complete physical examination.
6. Counsel females of childbearing potential to utilize effective contraception during the study.
7. Obtain approximately 19 mL of blood for hematology, biochemistry, serologic tests for viral hepatitis B and C and HIV in all volunteers and an extra 10 mL blood for β-HCG testing in females. Pre- and post-test counseling for blood borne viruses will take place as per existing SOP.
8. Obtain 45 ml blood for PBMC for CMI and serum for CMI assay for CMI immunity testing and for humoral immunity studies (anti-MSP2) by GIA and IFA. NB Sample can be collected Study Day 0 Pre-vaccination depending on laboratory availability.
9. Urine collection for dipstick testing.
10. Verify inclusion/exclusion criteria.
11. A screening number will be assigned to each volunteer.

## 6.3 Immunisation Procedure

Eligible participants will receive three immunisations 12 weeks apart. Vaccine/ Control will be prepared by the site pharmacist (or designee) according to Section 5.3 and provided to the vaccinator for administration within 30 minutes of being drawn into the syringe. A dose of 0.5 mL of the vaccine will be delivered by IM injection in the deltoid muscle of the arm after preparation of the site with alcohol. Successive vaccinations will be given in alternating arms.

## 6.4 Clinical Monitoring and Evaluation

The following sections provide a detailed listing of the procedures and studies to be performed in this protocol at designated time points. See **Appendix 1** for a tabular representation of study procedures. In addition to the procedures listed below, photographs of the injection site may be taken at baseline and/or on follow-up visits to better assess local reactogenicity.

Study Day 0 **(FIRST VACCINATION, visit 1)**

1. Verify that all applicable eligibility criteria have been met.
2. For females, obtain a urine sample for β-hCG testing. Ensure that test is negative before vaccinating; a positive test will exclude the volunteer from the trial.
3. Verify that Study Participation Informed Consent was obtained and a copy of this provided to the subject.
4. Medical Investigator to perform medical history and physical examination, to assess eligibility to enter vaccine phase.
5. Record vital signs (blood pressure, temperature, heart rate, and respiratory rate).
6. Obtain approximately 24 mL of blood for hematology (FBC and differential), biochemistry (electrolytes creatinine, liver function tests, CPK), and blood for study of humoral immune response by anti-MSP2 ELISA.
7. Administer the vaccine, and record which arm utilised.
8. Observe for at least 30 minutes after vaccination to evaluate for immediate adverse reactions. Vital signs will be repeated prior to leaving the clinic.
9. Education by study staff during 30-minute post-immunisation wait period describing proper use of digital thermometers, injection-site reaction measurement, and vaccine side-effect diaries. Study staff will also discuss signs and symptoms of potential AEs.
10. Distribute Days 0-6 diary card.
11. Record adverse events and concomitant medications.

Study Day 1 (Phone Call ONLY)

1. Phone call to inquire about diary card entries and injection site reactions since last visit.

Study Day 3 (visit 2)

1. Study staff to perform targeted history and examine injection site, emphasising examination of any acute complaints, with review by the medical investigator if required.
2. Record vital signs.
3. Obtain approximately 9 mL of blood for haematology (FBC and differential), and biochemistry (creatinine, liver function tests).
4. Record adverse events and concomitant medications.

Study Day 7 +/- 1 (visit 3)

1. Medical Investigator to perform medical history and physical examination including examination of injection site, emphasising examination of any acute complaints, to assess continued eligibility.
2. Record vital signs.
3. Collect Days 0-6 diary card and review completion. Refer any complaints to Medical Investigator for assessment.
4. Distribute Days 7-13 diary card.
5. Record adverse events and concomitant medications.

Study Day 14 +/- 2 (visit 4)

1. Medical Investigator to perform medical history and physical examination including examination of injection site, emphasising examination of any acute complaints and targetted history taking to elicit symptoms of muscle damage (myositis), as well as exercise history, emphasizing changes in pattern of regular exercise.
2. Record vital signs.
3. Collect Days 7-13 diary card and review completion. Refer any complaints to Medical Investigator for assessment.
4. Distribute Days 14-27 diary card.
5. Obtain approximately 14 mL of blood for haematology (FBC and differential), and biochemistry (creatinine, liver function tests, CPK).
6. Record adverse events and concomitant medications.

Study Day 21 +/- 3 (Phone call ONLY)

1. Phone call to inquire about diary card entries and injection site reactions since last visit.

Study Day 28 +/- 5 (1 month after FIRST vaccination, visit 5)

1. Study staff to perform targeted history and examine injection site, emphasising examination of any acute complaints, with review by the medical investigator if required.
2. Record vital signs.
3. Collect Days 14-27 diary card and review completion. Refer any complaints to Medical Investigator for assessment.
4. Obtain 55 ml of blood for collection of blood for humoral immunity studies by anti-MSP2 ELISA, and PBMC and serum for study of cell mediated immunity.
5. Record adverse events and concomitant medications.

Study Day 56 +/- 7 (2 months after FIRST vaccination, visit 6)

1. Medical Investigator to perform medical history and physical examination including examination of injection site, emphasising examination of any acute complaints, to assess continued eligibility.
2. Record vital signs.
3. Obtain approximately 9 mL of blood for haematology (FBC and differential), and biochemistry (creatinine, liver function tests).
4. Record adverse events and concomitant medications.

Study Day 84 +/- 7 (**SECOND vaccination, visit 7)**

1. Medical Investigator to perform medical history and physical examination, to assess continued eligibility to enter treatment phase.
2. For females, obtain a urine sample for β-hCG testing. Ensure that test is negative before vaccinating; a positive test will exclude the volunteer from the trial.
3. Record vital signs.
4. Obtain approximately 24 mL of blood for haematology, (FBC and differential), biochemistry (creatinine, liver function tests CPK) and approximately 55 mL blood for humoral immunity studies by anti-MSP2 ELISA.
5. Administer the vaccine in the alternate arm and record the arm utilised.
6. Observe for at least 30 minutes after vaccination to evaluate for immediate adverse reactions. Vital signs will be repeated prior to leaving the clinic.
7. Education by study staff during 30-minute post-immunisation wait period describing proper use of digital thermometers, injection-site reaction measurement tools and patient diaries. Study staff will also discuss signs and symptoms of potential AEs.
8. Distribute Days 0-6 diary card.
9. Record adverse events and concomitant medications.

Study Day 85 (1 day after SECOND vaccination, phone call ONLY)

1. Phone call to inquire about diary card entries and injection site reactions since last visit.

Study Day 87 (3 days after SECOND vaccination, visit 8)

1. Study staff to perform targeted history and examine injection site, emphasising examination of any acute complaints, with review by the medical investigator if required.
2. Record vital signs.
3. Obtain approximately 9 mL of blood for haematology (FBC and differential), and biochemistry (creatinine, liver function tests).
4. Record adverse events and concomitant medications.

Study Day 91 +/- 1 (7 days after SECOND vaccination, visit 9)

1. Medical Investigator to perform medical history and physical examination including examination of injection site, emphasising examination of any acute complaints, to assess continued eligibility.
2. Record vital signs.
3. Collect Days 0-6 diary card and review completion. Refer any complaints to Medical Investigator for assessment.
4. Distribute Days 7-13 diary card.
5. Record adverse events and concomitant medications.

Study Day 98 +/- 2 (14 days after SECOND vaccination, visit 10)

1. Medical Investigator to perform medical history and physical examination including examination of injection site, emphasising examination of any acute complaints and targetted history taking to elicit symptoms of muscle damage (myositis), as well as exercise history, emphasizing changes in pattern of regular exercise.

2. Record vital signs.

3. Obtain approximately 14 mL of blood for haematology (FBC and differential), and biochemistry (creatinine, liver function tests, CPK).

4. Collect Days 7-13 diary card and review completion. Refer any complaints to Medical Investigator for assessment.

5. Distribute Days 14-27 diary card.

6. Record adverse events and concomitant medications.

Study Day 105 +/- 3 (Phone call ONLY)
1. Phone call to inquire about diary card entries and injection site reactions since last visit.

Study Day 112 +/- 5 (28 days after SECOND vaccination, visit 11)

1. Study staff to perform targeted history and examine injection site, emphasising examination of any acute complaints, with review by the medical investigator if required.
2. Record vital signs.
3. Collect Days 14-27 diary card and review completion. Refer any complaints to Medical Investigator for assessment.
4. Obtain 55 ml of blood for collection of blood for humoral immunity studies by anti-MSP2 ELISA, GIA and IFA, and PBMC and serum for study of cell mediated immunity.
5. Record adverse events and concomitant medications.

Study Day 140 +/- 7 (56 days after SECOND vaccination, visit 12)

1. Medical Investigator to perform medical history and physical examination including examination of injection site, emphasising examination of any acute complaints, to assess continued eligibility.

2. Record vital signs.

3. Obtain approximately 9 mL of blood for haematology (FBC and differential), and biochemistry (creatinine, liver function tests).

4. Record adverse events and concomitant medications.

Study Day 168 +/- 7 (**THIRD VACCINATION, visit 13**)

1. Medical Investigator to perform medical history and physical examination, to assess continued
eligibility to enter treatment phase.

2. Obtain approximately 24 mL of blood for haematology, (FBC and differential), biochemistry (creatinine, liver function tests CPK), and humoral immunity studies by anti-MSP2 ELISA.

3. For females, obtain a urine sample for β-hCG testing. Ensure that test is negative before vaccinating; a positive test will exclude the volunteer from the trial.

4. Record vital signs.

5. Administer the vaccine in the alternate arm.

6. Observe for at least 30 minutes after vaccination to evaluate for immediate adverse reactions. Vital signs will be repeated prior to leaving the clinic.

7. Education by study staff during 30-minute post-immunisation wait period describing proper use of digital thermometers, injection-site reaction measurement tools, and patient diaries.  Study staff will also discuss signs and symptoms of potential AEs.

8. Distribute Days 0-6 diary card.

9. Record adverse events and concomitant medications

Study Day 169 (1 day after THIRD vaccination, Phone call ONLY)

1. Phone call to inquire about diary card entries and injection site reactions since last visit.
 
Study Day 171 (3 days after THIRD vaccination, visit 14)

1. Study staff to perform targeted history and examine injection site, emphasising examination of
any acute complaints, with review by the medical investigator if required.

2. Record vital signs.

3. Obtain approximately 9 mL of blood for haematology (FBC and differential), and biochemistry (creatinine, liver function tests).

4. Record adverse events and concomitant medications.

Study Day 175 +/- 1 (7 days after THIRD vaccination, visit 15)

Medical Investigator to perform medical history and physical examination including examination of injection site, emphasising examination of any acute complaints, to assess continued eligibility.

Record vital signs.

Collect Days 0-6 diary card and review completion. Refer any complaints to Medical Investigator for assessment.

Distribute Days 7-13 diary card.

Record adverse events and concomitant medications.

Study Day 182 +/- 2 (14 days after THIRD vaccination, visit 16)

1. Medical Investigator to perform medical history and physical examination including examination of injection site, emphasising examination of any acute complaints and targetted history taking to elicit symptoms of muscle damage (myositis), as well as exercise history, emphasizing changes in pattern of regular exercise.
2. Record vital signs.
3. Collect Days 7-13 diary card and review completion. Refer any complaints to Medical Investigator for assessment.
4. Distribute Days 14-27 diary card.
5. Obtain approximately 14 mL of blood for haematology (FBC and differential), and biochemistry (creatinine, liver function tests, CPK).
6. Record adverse events and concomitant medications.

Study Day 189 +/- 3 (Phone call ONLY)
1. Phone call to inquire about diary card entries and injection site reactions since last visit.

Study Day 196 +/- 5 (28 days after third vaccination, visit 17)

- 1. Study staff to perform targeted history and examine injection site, emphasising examination of any acute complaints, with review by the medical investigator if required.
  2. Record vital signs.
  3. Collect Days 14-27 diary card and review completion. Refer any complaints to Medical Investigator for assessment.
  4. Obtain 55 ml of blood for collection of blood for humoral immunity studies by anti-MSP2 ELISA, GIA and IFA, and PBMC and serum for study of cell mediated immunity.

5. Record adverse events and concomitant medications.

Study Day 336 +/- 14 (6 months after THIRD vaccination, visit 18)

1. Medical Investigator to perform medical history and physical examination including examination of injection site, emphasising examination of any acute complaints.
2. Record vital signs.
3. Obtain 55 ml of blood for collection of blood for humoral immunity studies by anti MSP2 ELISA, GIA and IFA, and PBMC and serum for study of cell mediated immunity.

## 6.5 Volunteer Symptom Diary

Volunteers will be asked to keep daily symptom diaries recording oral temperature as well as pain/ tenderness, redness, swelling at the injection site and any systemic signs or symptoms for 14 days following each immunization (as two diary cards, Day 0-6, Day 7-13) and a daily symptom diary recording local injection site symptoms for Days 14-27 (third diary card Day 14-28). The size of any injection-site reaction will be measured using a standardized clear plastic measurement device and recorded in the study volunteer symptom diary.

## 6.6 Laboratory Testing

Using standard techniques, laboratory testing will be performed by the certified Clinical Laboratory.

1. Full blood count, including white blood cell differential
2. Serum creatinine
3. AST and ALT, Creatinine Phosphokinase (CPK)
4. HIV and Hepatitis B sAg, Hepatitis C Antibody test
5. βhCG (Females only)
6. Urinalysis (in the event of an abnormal urine dipstick test full microurine tests will be undertaken)

Urine β-hCG testing will be performed at the clinical trial site using an approved urine pregnancy test kit. Urinalysis will be tested by urine dipstick testing at the trial site using an approved product. Microscopic examination will be undertaken only in the case of clinically significant abnormality by dipstick.

## 6.7 Immunologic Testing

Immunology studies will be performed at the three locations:

WEHI, J. Beeson’s Laboratory – antibody reactivity (ELISA, immunofluorescence, Western blots) and growth inhibition assays using dialysed serum.

NIH, C. Long’s Laboratory – Growth inhibition assays using purified IgG.

QIMR/QHPS - Cellular Immunology

Blood samples collected from participants will be processed and aliquoted according to specific laboratory instructions. The trial site will securely store all serum/plasma samples at -80ºC until shipment. Frozen sera/plasma will be shipped to the Laboratories on dry ice in batches. An inventory of each shipment will be maintained by both the trial site and the analysing Laboratory. Peripheral Blood Mononuclear Cells (PBMC) will be prepared according to the analytical laboratory procedures and transferred to the laboratory for assay preparation.

### 6.7.1 Antibody Assays

### 6.7.1.1 Enzyme Immunoassays (ELISA)

Antibody levels to the MSP2 antigens will be measured in serum by ELISA using established protocols. The MSP2 antigens for these assays will be provided by CSL Limited. Each sample will be tested in duplicate against 3D7 and FC27 MSP2 alleles. A titration of a reference positive control serum will be included in every assay. Total IgG, IgM and IgG isotypes will be measured. In addition to MSP2-3D7 and MSP2-FC27, serum samples may also be tested against MSP2 proteins with other sequences.

### 6.7.1.2 Immunofluorescence microscopy

A selection of samples that were found to be positive to MSP2 by ELISA will be tested for reactivity to the surface of merozoites using confocal immunofluorescence microscopy. *In vitro* cultures of *P. falciparum*-infected erythrocytes at the schizont stage will be smeared on glass slides, fixed, and probed with ELISA-positive serum from vaccinnees and from recipients of ISA720 Control.

### 6.7.2 Western blotting of parasite protein extracts

A selection of positive serum samples, and serum from participants receiving ISA720 Control vaccine, will be tested for immunoreactivity of MSP2 in protein extracts of *P. falciparum* by Western blot. *P. falciparum*-infected erythrocytes at the schizont stage and a protein extract prepared using established procedures. The protein extract containing MSP2 will be run on a SDS-PAGE gel followed by Western transfer and blotting with serum samples. Rabbit antibodies raised against MSP2 will act as a positive control.

### Growth Inhibition Assay

The GIA is designed to determine whether anti-MSP2 antibodies obtained from a vaccinee can inhibit merozoite invasion of red cells. This will be performed using two different established approaches, in C. Long’s laboratory, NIH (MVI-supported GIA Reference Center), and J. Beeson’s Laboratory, WEHI (34).

Synchronized mature blood-stage parasites from the 3D7 and FC27 lines are incubated with purified IgG (Long Laboratory) or dialysed serum (Beeson Laboratory) from volunteer sera *in vitro*. The number of infected red cells will be determined by assaying parasite lactate dehydrogenase activity (Long Laboratory) or by flow cytometry (Beeson Laboratory). Growth inhibitory activity is defined as the percentage reduction in the number of newly invaded red cells (determined by parasitemia) by post vaccination serum/IgG compared to serum/purified IgG from Day 0 samples or samples from recipients of the ISA720 Control. In addition to 3D7 and FC27, serum samples may also be tested against parasites expressing MSP2 with other sequences

### Cell Mediated Immunity (CMI)

T-Cell Proliferation and cytokine response

The T-cell proliferation will define the cellular immune response to the vaccine candidate. The assay involves *in vitro* culture of PBMC with vaccine antigen and appropriate controls. T-Cell proliferation and cytokine responses will be described in the Laboratory Reference methods.

## 6.8 Use, Storage, and Tracking of Specimens and Data

Immunological samples and data collected under this protocol will be used to study malaria and possible adverse reactions to vaccination. No genetic testing will be performed. Access to research samples will be limited using either a locked room or a locked freezer. Samples and data will be stored using codes assigned by the investigators or their designees. Data will be kept in password-protected computers. Only investigators or their designees will have access to the samples and data.

Aliquots of serum/plasma samples will be stored at both QIMR and WEHI. Samples will be tracked using sample logs. The research use of stored, unlinked, or unidentified samples may be exempt from the need for prospective HREC/IRB review and approval. Exemption requests will be submitted in writing to the relevant Human Research Ethics Committees authorised to determine whether a research activity is exempt.

Participants may decide at any point not to permit their samples to be stored. In this case, the principal investigator or the laboratory nominee will destroy all known remaining samples and report what was done to both the subject and to the IRB/HREC’s. This decision will not affect the subject’s participation in this protocol or any other protocols.

# 7.0 Adverse Events Monitoring and Reporting

## 7.1 Definitions

### 7.1.1 Adverse Event (AE)

An adverse event (AE) includes any noxious, pathological, or unintended change in anatomical, physiological or metabolic functions as indicated by physical signs, symptoms and/or laboratory-detected changes occurring in any phase of the clinical study, whether associated with the study vaccine or ISA720 Control, and whether or not considered vaccination related. This includes an exacerbation of pre-existing conditions and intercurrent illnesses. Unchanged pre-existing conditions will not be included as an adverse event. All AEs must be graded for intensity and relationship to the investigational vaccine as described in **Sections 7.2.2** and **7.2.3**.

### 7.1.2 Serious Adverse Event (SAE)

An SAE is an AE, whether considered related to the investigational vaccine or not, meeting one of the following conditions:

1. Death during the period of protocol-defined surveillance.
2. Life threatening: defined as an event that places a subject at immediate risk of death at the time of the event and does not refer to an event that hypothetically might have caused death were it more severe.
3. Hospitalisation during the period of protocol-defined surveillance: defined as at least an overnight stay in the hospital or emergency ward for treatment that would have been inappropriate if administered in the outpatient setting.
4. Results in a congenital anomaly or birth defect.
5. Results in a persistent or significant disability or incapacity: defined as a substantial disruption of the study participant’s ability to carry out normal life functions.
6. Any other important medical event that may not result in death, be life threatening, or require hospitalization, may be considered a serious AE when, based upon appropriate medical judgment, the event may jeopardize the subject and may require medical or surgical intervention to prevent one of the outcomes listed above.

## 7.2 Assessment of Adverse Events

### 7.2.1 Identification of AEs

Assessment of safety will include clinical observation and monitoring of hematological, chemical and immunologic parameters. Safety will be evaluated by monitoring of volunteers for local and systemic adverse reactions during the course of the trial. Volunteers will be closely observed in the clinic for approximately 30 minutes after each vaccination and contacted by telephone on Day 1 and 21 after each vaccination to inquire about reactions. Additionally, volunteers will return to the clinic on Days 3, 7, 14 and 28 following each vaccination for clinical assessments. Clinical staff must accurately measure the total affected area of any volunteer presenting with erthyema, swelling and induration with a ruler or tape measure. Measurement must be documented at each visit until resolution. Clinical staff may elect to photograph injection site reactions over time for any volunteer. Clinical staff must apply the Brighton’s Collaboration Assessment Tool on any nodules or suspected nodules or to differentiate induration from a nodule.

For 13 days after each immunisation, volunteers will be asked to keep daily diaries of symptoms, recording oral temperature, as well as a subjective assessment of the extent of induration, erythema, pain/ tenderness at the site of injection, and any systemic signs and symptoms. The size of any injection-site reaction will be measured using a standardized clear plastic measurement device and recorded in the volunteer symptom diary. A third diary card will be given at the 14 day visit following each vaccination for the volunteer to record only injection site reactions. (Days 14-28).

At each clinic visit to Day 28, clinic staff will review the volunteer diaries, confirm the symptoms experienced and record AEs as appropriate in the source document. Individual symptom reports of pain, tenderness and muscle ache at injection site will be recorded as the AE term of pain at injection site.

All AEs will be graded for intensity and relationship to study product. Reactions will be graded as described in **Appendix 2** in this protocol. A study clinician will be available by telephone or pager 24 hours a day during the study evaluation period. Should a volunteer call a study clinician to report an adverse event, it will be determined at that time if an extra visit(s) will be scheduled, and/or appropriate medical advice will be provided. Additionally, all calls will be documented in the volunteer’s study chart, and discussed with the Principal Investigators.

All local and systemic reactions not meeting the criteria for “serious adverse events” will be captured on the appropriate case report form (CRF). These events will be followed to adequate resolution.

### 7.2.2 Determination of Severity

Severity of AEs will be assessed by the investigator as described in **Appendices 2, 3, and 4**. AEs not included in **Appendices 3 and 4** will be graded for severity using the following definitions:

Grade 0 (None):

Grade 1 (Mild): No effect on activities of daily living.

Grade 2 (Moderate): Partial limitation in activities of daily living (can complete ≥ 50% of baseline), or treatment given.

Grade 3 (Severe): Activities of daily living limited to < 50% of baseline, or medical evaluation required.

Grade 4 (Life- Extreme limitation in activity, significant assistance required;

threatening): immediate medical intervention or therapy required to prevent death.

Grade 5: Death.

Intensity of solicited AEs will be assessed by the investigator as described in **Appendix 2.** All laboratory AEs will be graded in severity following the toxicity table in **Appendix 3**. Unexpected adverse events not described in the protocol will be graded according to the common toxicity table in **Appendix 4**.

The maximum observed intensity reported for any AE is recorded for the entire duration of the AE.

### 7.2.3 Association with Receipt of the Study Vaccine

All AEs will have their possible relationship to study vaccine assessed using the following definitions:

Definite: Clear-cut temporal association, and no other possible cause.

Probable: Clear-cut temporal association and a potential alternative etiology is not apparent.

Possible: Less clear temporal association; other etiologies also possible.

Unlikely: Temporal association between the AE and the vaccine or the nature of the event is such that the vaccine is not likely to have had any reasonable association with the observed illness/event (cause and effect relationship improbable but not impossible).

Not Related: The AE is completely independent of vaccine administration; and/or evidence exists that the event is definitely related to another etiology.

The degree of certainty with which an AE can be attributed to administration of the study vaccine will be determined by how well the event can be understood in terms of one or more of the following:

1. The event being temporally related with vaccination or reproduced on re-vaccination.
2. A reaction of similar nature having previously been observed with this type of vaccine and/or formulation.
3. The event having often been reported in the literature for similar types of vaccines.

All local (injection-site) reactions will be considered causally related to vaccination.

## 7.3 Adverse Event Reporting

All SAEs will be reviewed by a study physician, recorded on the appropriate SAE form, and followed through to resolution by a study physician. All SAEs will be reported by telephone, fax, or email within 1 working day of notification of the SAE occurrence to all of the following:

- Queensland Institute of Medical Research-Human Research Ethics committee
  (QIMR-HREC): Phone: 61-7 3362 0117, Fax: 61-7 3362 0111.
- Western Institutional Review Board (WIRB®): PATH-MVI will report adverse events to WIRB.
- WEHI Walter and Eliza Hall Institute – Human Research Ethics Committee, 1G Royal Parade, Parkville 3050, Victoria, Australia.

Following notification from the Principal Investigators, the sponsor (QIMR), will report events that are both serious and/or unexpected that are possibly, probably or definitely related to the vaccine, to the TGA within the required timelines: fatal and life-threatening events within 7 calendar days (by phone or fax) and all other SAEs in writing within 15 calendar days. In addition, the Principal Investigators will notify the QIMR nominated CRO monitor within 1 working day of notification of the SAE occurrence, who will in turn notify MVI. MVI will then notify the WIRB.

- Australian TGA Sponsor (Queensland Institute of Medical Research [QIMR]):
  Phone: 61-7-3362 0222, Fax: 61-7-3362 0109
- Funding Sponsor (PATH-MVI): Phone: +1-240-395-2700, Fax: +1-240-395-2591 or by Email: emalkin@malariavaccine.org

All SAEs not listed as possibly, probably or definitely related will be reported to the IRB/ HREC at least annually in a summary format as required by the institution. All local and systemic reactions not meeting the criteria for “serious adverse events” will be captured on the appropriate case report form (CRF). These events will be followed to resolution or stablisation. (Allow for 30 days after last study visit /or conclusion of the study.

## 7.4 Adverse Event Monitoring

### 7.4.1 Local Medical Monitor

An independent local Medical Monitor has been appointed for oversight of safety in this trial. The local Medical monitor will be available to advise the investigators on trial-related medical questions or problems, and act as a representative for the volunteers’ welfare. Additionally, the local Medical Monitor may ask to convene a safety monitoring committee (SMC) meeting for review of any safety issue or adverse event. The Medical Monitor will review all SAEs.

### 7.4.2 Safety Monitoring Committee

The sponsor (QIMR) in consultation with MVI will select three independent monitors to form the Safety Monitoring Committee to advise the site and the study investigators on the trial safety. The SMC will be independent of sponsors and the clinical trial site. The SMC’s primary responsibility will be to monitor volunteer safety. The SMC will meet at the nominated schedule (Table 1) following the immunizations of the 10 µg, 40 µg and 80 ug dose groups to approve further vaccination in selected cohorts and dose escalation, and whenever the need should arise. The SMC will review cumulative safety data for evidence of study-related AEs, adherence to the protocol, and factors that may affect outcome or study data such as protocol violations and losses to follow-up. At each meeting the SMC will make a recommendation regarding the safe continuation of the study. If no stopping criteria are met (**Section 7.5**), with the approval from the SMC the trial will proceed.

The Principal Investigators are responsible for ensuring that the SMC is aware of all new safety information.

## 7.5 Criteria for Placing the Study on Hold

If a dose of vaccine is considered significantly reactogenic (see below), additional vaccinations will be suspended until a thorough review by the independent Medical Monitor and SMC and discussion between the Principal Investigators, the Medical Monitors, and Sponsors. Resumption of vaccinations will start only if all parties agree to a resumption of vaccination. The communications from the SMC will subsequently be forwarded by the investigators to the respective IRBs.

The following criteria will be used to define significant reactogenicity:

1. One or more volunteers experience an SAE (as defined in **Section 7.1.2** in this protocol) that is determined to be possibly, probably, or definitely related to the vaccine (as defined in **Section 7.2.3** in this protocol), **OR**
2. One or more volunteers experience anaphylaxis that is probably or definitely related to the vaccine, **OR**
3. Occurrence of any severe clinical illness in one or more volunteers that is not explained by another diagnosis unrelated to vaccination, **OR**
4. Two or more volunteers experience a Grade 2 or higher laboratory abnormality, with the exception of CPK. In the case of CPK, if in the absence of other explanation (vigorous unaccustomed exercise) one volunteer experiences a Grade 3 or higher elevation of CPK or if two or more volunteers experience a Grade 2 elevation of CPK.
5. One volunteer experiences a Grade 3 clinical AE that is determined to be probably or definitely related to the vaccine, with the exception of isolated Grade 3 local erythema or swelling.
6. The principal investigator (PI) may, using discretion, ask for the study to be placed on hold and an SMC meeting to be held for any single event or combination of multiple events which, in professional opinion, jeopardize the safety of the participants or the reliability of the data.

### 7.5.1 Criteria for Stopping an Individual Volunteer’s Further Vaccination

A. *Local Reactions*: Investigator discretion

B. *Systemic AEs*: Individual participants will be withdrawn from further immunisation if they develop a Grade 3 AE beginning within two days after immunisation and persisting at Grade 3 ≥48 hours if the AE is malaise, myalgia, fatigue, fever, headache, or joint pain, or if the subject has any other Grade 3 AE considered to be associated with vaccination, or if they have any vaccine-related SAE.

C. *Laboratory AEs*: Individual participants will be withdrawn from further immunisation for any Grade 2 laboratory AE that lasts for ≥48 hours or any Grade 3 laboratory AE considered possibly, probably or definitely associated with immunisation.

### 7.5.2 Process for Study Discontinuation

If the Principal Investigators, Sub-Investigators, Medical Monitor or sponsors have any further safety concerns, the SMC can be convened by the sponsors to review the safety data. The committee will provide verbal and hard copy written reports to the investigators and sponsors, who will then forward to the appropriate HREC/IRBs. Findings of a serious and immediate nature including any recommendations to modify or discontinue all or part of the study must be reported immediately in verbal and written form. Under circumstances where there are safety concerns that warrant no immunisation of a cohort, no further immunisations will be performed, and the Sponsors and all IRB/HRECs and Regulatory authorities will be promptly notified. All immunized participants will be followed for safety until resolution of their AEs or until at least 30 days after the last immunisation.

# 8.0 Data Collection and Monitoring

## 8.1 Source Documentation

Complete source documentation (laboratory test reports, hospital or medical records, etc.) is required for every study subject for the entire duration of the study. Clinical Report Forms (CRFs) and volunteer symptom diaries will be used to record data for participants enrolled in the study. The Investigator is responsible for the accuracy and completeness of the data reported to the Sponsor in the CRFs and diaries. Data reported in the CRFs derived from source documents should be consistent with source documents or the discrepancies should be explained.

## 8.2 Study Documentation

Study-related documentation will be completed as required by the HREC/IRBs, the Sponsor, ICH Guidelines, and regulatory authorities and maintained at the trial site. Continuing review documentation will be submitted by the Investigators to the HREC/IRBs as specified by each committee. An annual report will be submitted by the investigator to the HREC/IRB on the anniversary approval date for MSP2/ISA720 malaria vaccine went into effect. These reports will provide a brief description of the progress of the investigation as outlined in NHMRC National Statement and will include any revisions of the protocol.

The Investigator will maintain adequate records of the disposition of the investigational product, including dates, quantity and use by participants. If the study is terminated, suspended or completed, final disposition of unused vaccine supplies will be determined by Q-Pharm, in conjunction with the Sponsors.

In addition to the study-related documentation required by the regulatory authorities, the Sponsor (QIMR) will also submit a final study report to the Funding Sponsor (MVI). The final study report will be completed after the safety and immunogenicity data from all study visits have been compiled and analysed. A Clinical Safety Report will be prepared by the CRO and submitted to the Sponsors after database lock and statistical analysis is completed. The Sponsor (QIMR) will prepare the final Clinical Study Report.

## 8.3 Access to Study Documentation:

The principal investigator will permit trial-related monitoring, audits, IRB/ethics review, regulatory inspections (national or international) by providing direct access to source/data documents when requested.

## 8.4 Clinical Database

In addition to the primary CRF maintained on paper, an electronic clinical database will be built to facilitate management of clinical data, and to automate data quality processes. The database will be built and managed in accordance with FDA 21CFR11. Data from the paper CRFs will be double entered into the database, and reconciled. Automated edit checks for data quality will be programmed. Data items failing such quality checks will be queried to the site. 10 % of data base records will be audited for quality assurance purposes.

Full details of the data management process, including the data dictionary, edit checks and query handling will be documented in a data management plan.

## 8.5 Retention of Records

Trial-related documents will be maintained by the site for a period of 2 years after final marketing approval of the vaccine, or for 2 years following the formal discontinuation of clinical development of the product or per local law regulations, whichever is longer. The Sponsor is required to inform the Investigator as to when such documents need no longer be retained. Storage of all trial-related documents will be such that confidentiality will be strictly maintained.

## 8.6 Protocol Revisions

No revisions to this protocol will be permitted without documented approval from both the Sponsors and the IRBs that granted the original approval for the study. Furthermore, in the event of a medical emergency, the Investigator shall perform any medical procedures that are deemed medically appropriate. The Investigator must notify the Sponsor of all such occurrences. Any change to the protocol will be submitted to the participating IRBs as a protocol amendment and changes not affecting risk to volunteers may be expedited, as appropriate.

## 8.7 Clinical Investigator’s Brochure

Investigators will receive the current version of the Clinical Investigator’s Brochure, which comprehensively describes all the available preclinical and clinical experience with the experimental vaccine and adjuvant. If relevant new information becomes available during the course of the trial, the Investigators will receive a revised Investigator’s Brochure or an amendment to the current version.

## 8.8 Study Monitoring

The Sponsor (QIMR) will monitor through delegated responsibility via a CRO (Clinical Network Services), all aspects of the study, with respect to current Good Clinical Practices (GCP), and ICH Guidelines for compliance with applicable government regulations. Prior to the start of the study, the Investigator will be informed of the frequency of monitoring visits and will be given reasonable notification prior to each visit. The objectives of a monitoring visit will be to verify the prompt reporting of SAEs, to check the availability of the signed Informed Consent, GCP adherence to the protocol, and to compare CRFs with source data for completeness and accuracy. During the monitoring visit, the Investigator (and/or designee) and other study personnel should be available to discuss the study. Study documents must be available for review throughout the course of the study.

# 9.0 Statistical Considerations

## 9.1 General Design

The goal of this Phase 1 vaccine trial is to demonstrate safety and immunogenicity of MSP2-C1/ISA720 malaria vaccine in healthy adult human volunteers.

### 9.1.1 Description of the Statistical Methods to Be Employed

The purpose of this trial is to estimate event rates and patterns of adverse events for safety as well as immune responses for the three different doses of the study vaccine. This section briefly describes the statistical methods to be used. A detailed Statistical Analysis plan will fully describe the methods and will be finalized prior to lock of the study database. The analytic plan will discuss the planned approaches to missing data.

Any deviations from the statistical analysis plan will be documented and justified in the statistical report.

Estimates will be presented with their 95% confidence intervals. Analysis will involve both descriptive exploratory methods, and hypothesis driven significance testing. In particular, the immunologic response and the adverse events for each of the 45 volunteers (Active versus ISA720 Control) will be presented as individual graphs.

### 9.1.2 Analyses for Primary Objective (safety)

To assess the safety and tolerability of three immunisations at three dosage levels of the MSP2-C1/ISA720 vaccine in healthy malaria-naive volunteers

The incidence of AE and SAEs will be tabulated by treatment group (with the pooled ISA720 Control patients representing one group), System Organ Class and Preferred Term. Incidence in each treatment group will be compared with that in the pooled ISA720 Control group using Fisher's exact test (correcting for multiple comparisons across AEs using Holm's method (37). Separate assessments of systemic and local reactions will be performed. The overall number and percentage of volunteers with at least one AE (and SAE) will be tabulated after each vaccination and over the entire study period. In addition, longitudinal comparisons will be made for each treatment group using Lidell’s exact version of McNemar’s test. The incidence, intensity, and relatedness to vaccination of individual symptoms will be calculated for each dose and study arm. The incidence, intensity and relatedness to vaccination will be calculated for each treatment group.

If any clinically significant deviations from normal occur in routine laboratory test results and/or vital signs, they will be analyzed using a longitudinal linear mixed model, with Treatment Group and Time as fixed effects, and subject and times within subject as random effects. An appropriate covariance structure will be selected for the longitudinal error term. These analyses will be repeated for change from baseline values, where the baseline is given by pre-dose values.

### 9.1.3 Analyses for Secondary Objectives (Immunogenicity)

### 9.1.3.1 Characterise the humoral and cellular immune responses to the MSP2-C1/ISA720 vaccine:

Anti-MSP2 antibodies (anti-MSP2 3D7 & anti-MSP2 FC27- Total and Ig G subtype) will be measured by ELISA for each serum sample. Immunogenicity responses will be described by vaccine and dose group, over time. Individual responses will be described over time and stratified by dose cohort. Antibody titres will be measure on Days 0, 28, 84, 112, 168, 196, and 336). The primary immunogenicity endpoint will be evaluation of sera taken at 28 days after each vaccination, and at six months after the final vaccination as listed in the schedule of visits (Appendix 1).

Antibody responses will be analysed using a longitudinal linear mixed model, with MSP2 serotype, Dose and Time as fixed effects, and Subject and Times within Subject as random effects. An appropriate covariance structure will be selected for the longitudinal error term. Because of the small sample size of this study, statistical tests will be performed without correction for multiplicity. A nominal Type I error rate of 10 percent will be used. Parameter estimates will be obtained using the REML algorithm (38). Contrasts will be generated to evaluate MSP2 serotype and Dose effects at each time point. Formal statistical tests will assess whether the response is monotone (low dose < medium dose < high dose) for the 3 doses of vaccine being tested to look for a dose response.

The vaccine dose level which induces the highest concentrations of MSP2 specific IgG will be determined graphically.

Additionally, the cell mediated responses (T cell proliferation, cytokines) will be performed on Days 0, 28, 112, 196, and 336. T cell proliferation and cytokines will be analysed in the same way, using a longitudinal linear mixed model. Analyses will include (but not be limited to) contrasts to examine difference between MSP2 serotypes and Dose levels at each time point. All of these analyses will be repeated using pre vaccination (day 0) values as a covariate.

The following parameters will be summarised by simple descriptive statistics:

Parameter 1: The ability of the vaccine induced antibody to inhibit parasite growth as measured by the in vitro growth inhibition assay (GIA)

Parameter 2: The relationship between anti-MSP2 antibody levels and degree of in vitro parasite growth inhibition.

Graphs will display growth inhibition expressed as a percent of inhibition comparing test sera to pre-immune sera. Depending on the distribution of the data, parametric or non-parametric methods will be used to compare inhibition as a function of dose, and serotype.

Should the need arise for terminating the study early, the investigative team will discuss with the SMC the reason for termination and determine which study questions can be addressed in an unbiased manner with the available data. The available data will be analyzed and interpreted in light of early termination. Deviations from the statistical plan will be reported in the study report.

### 9.1.4 Sample Size

This study is powered to provide sufficient safety data on an adult population living in an area without malaria. A group size of 12 volunteers per dose will receive the MSP2-C1/ISA720 malaria vaccine and a total of 9 participants, 3 per dose cohort will receive ISA 720 Control vaccine as a control. This will give a probability of 0.80 for detecting one or more serious or severe AEs that occurred with a probability of 0.15 per volunteer.

# 10.0 Protection of Human Subjects

## 10.1 Institutional Review Board/Human Research Ethics Committee

The study will be conducted in accordance with the principles of the Declaration of Helsinki (Recommendations guiding Medical Doctors in Biomedical Research Involving Human Subjects), and with the NH&MRC National Statement on Ethical Conduct in Research Involving Humans (2007). The conduct of the study will be in accordance with the Notes for Guidance on Good Clinical Practice (CPMP/ICH/135/95), as adopted by the Australian Therapeutic Goods Administration (2000).

The Investigator will be responsible for obtaining IRB/HREC approval for the study. Before the start of the study, the appropriate documents (including the Protocol, Investigator’s Brochure, Informed Consent Form, information sheets, Diary Cards and advertisements) will be submitted to the IRBs/HRECs. A copy of the study approval (including approval of the informed consent form) is to be maintained in the Investigator’s study document binder and a copy will be supplied to the Sponsor. During the study, the Investigator is responsible for providing the IRB/HRECs with all documents subject to review (i.e., Protocol Amendments, informed consent form updates, advertisements, and any written information that may be provided to the subject). Annual reports on the progress of the study will be made to the IRBs/HRECs by the Investigator in accordance with IRB/HREC guidelines and government regulations.

## 10.2 Informed Consent

In obtaining and documenting informed consent, the Investigator must comply with the applicable regulatory requirements, Good Clinical Practices, ICH Guidelines, and ethical principles. The written informed consent form must be approved by all IRBs/HRECs prior to its use.

Participants will be fully informed of the nature of the study, the properties and side effects of the investigational products, and all relevant aspects of study procedures. Subject information is provided predominantly by a Medical Investigator during recruitment. Participants will receive a copy of the ‘Privacy Information and Informed Consent Form for Screening’ (Appendix 5) and the ‘Information for Volunteers and Consent Form for Study Participation’ (Appendix 6). They may ask questions of a Medical Investigator or the Clinic Staff at any time.

The recruitment procedures consent form will be signed and dated by the volunteers in the presence of an investigator. The study participation consent form will be signed and dated by the volunteers in the presence of an investigator.

## 10.3 Risks

Risks to the volunteers are associated with venipuncture and with immunisation. These risks are outlined below.

Female participants will be counseled on the unknown risk of the MSP2-C1/ISA720 vaccine to the foetus and will be advised to use adequate birth control methods from screening through the entire trial duration. Any female participant interested in contraceptive methods will be referred to the local health center or family planning services for evaluation and institution of an appropriate contraceptive method.

### 10.3.1 Venipuncture

Risks occasionally associated with venipuncture include pain and bruising at the site of venipuncture, lightheadedness, and syncope (rarely). Volunteers will be advised not to donate blood during the study and for 30 days after the study ends.

### 10.3.2 Immunisation

Possible local vaccine reactions include pain, swelling, erythema, induration, nodule formation, limitation of limb movement for several days, lymphadenopathy, or pruritis at the injection site. Delayed type local injection site reactions may be seen with the Montanide® ISA720 adjuvant. Systemic reactions such as fever, chills, headache, fatigue, malaise, myalgia and joint pain may also possibly occur. Immediate hypersensitivity reactions including urticaria, anaphylaxis or other IgE mediated responses are possible as with any vaccine. As with any investigational vaccine, there is a theoretical possibility of risks about which we have no present knowledge. Volunteers will be informed of any such risks should further data become available.

### 10.3.3 Abnormal muscle enzyme levels

In two volunteers in cohort 2A, significant abnormalities in biochemical tests undertaken around the day 14 safety review indicated that there is laboratory evidence of transient damage to skeletal muscle, with elevations in CPK > 5,000. While such abnormalities can be observed after vigorous exercise, in one of these two subjects no such activity was reported. Such findings, to our knowledge have never been observed at this timepoint following vaccinations. To date, we do not have a clear physiologic process that can clearly define the etiology. Therefore, for the remainder of the trial it is planned to undertake more close evaluations for history of, symptoms of and biochemical markers of muscle damage around this time period (exercise history, symptoms of myositis, elevations in CPK and close monitoring of transaminase levels).

## 10.4 Precautions Taken to Minimize Risks

As outlined above, the participants will be monitored closely during their participation in this study. The study vaccines have been produced according to cGMP. The vaccines will be administered by experienced investigators or study nurses with drugs and equipment for the treatment of anaphylaxis and other potential adverse reactions readily available. All vaccine doses will be given by IM injection to minimize injection site reactions such as pain.

## 10.5 Benefits

Volunteers will not receive any direct benefit from the vaccinations administered in this study. It is hoped that information gained in this study will contribute to the development of a safe and effective malaria vaccine.

## 10.6 Confidentiality

Participants will be informed (Appendix 5) that their data are held on file by Q-Pharm, that these data may be viewed by staff of Q-Pharm (including, where necessary, staff of Q-Pharm other than the named investigators), and that data may also be sighted by the sponsors’ monitor on behalf of the sponsor and by external auditors on behalf of either the sponsors or regulatory agencies.

All study-related information will be stored securely at the study site. All participant information will be stored in locked file cabinets in areas with access limited to study staff. All laboratory specimens, reports, study data collection, process and administrative forms will be identified by coded number only to maintain participant confidentiality. All computer entry will be done by coded number only, and all local databases will be secured with password-protected access systems. Forms, lists, logbooks, appointment books and any other listings that link participant ID numbers to other identifying information will be stored in a separate, locked file in an area with limited access.

The participants will similarly be informed that a report of the study will be submitted to the sponsors and may also be submitted to government agencies and perhaps for publication, but that they will only be identified in such reports by their study identification number and perhaps their gender and age.

## 10.7 Compensation

Volunteers who complete the study will be paid $1050 compensation for their participation in completing the study. The total payment will be divided over the course of the study with the final payment following the last visit.

Reserve participants who do not participate in the study will be paid $150 compensation for the inconvenience associated with their attendance for screening and for their attendance on the dosing day of vaccination, in case they are required to participate.

Participants who withdraw or are withdrawn from the study will be compensated on a fractional basis for their involvement unless they are withdrawn as a consequence of their misconduct and/or non-compliance.

## 10.8 Liability/Indemnity/Insurance

The study sponsor, QIMR, will ensure sufficient insurance is available to enable it to indemnify and hold the investigator(s) and relevant staff, including medical monitor as well as any hospital, institution, ethics committee or the like, harmless from any claims for damages for unexpected injuries, including death, that may be caused by the study drug but only to the extent that the claim is not caused by the fault or negligence of the participants or investigator(s). QIMR and PATH (a named party to the policy) agree to adhere to the guidelines of Medicines Australia for injury resulting from participation in a company sponsored trial, including the provision of “No-fault clinical trial insurance”.

# 11.0 References

1. WHO and UNICEF World Malaria Report 2005.

2. Smythe JA, Coppel RL, Brown GV, et al. (1988). Identification of two integral membrane proteins of Plasmodium falciparum. *Proc Natl Acad Sci. USA 85:* 5195-5199.

3. Gerold P, Schofield L, Blackman MJ, et al. (1996) Structural analysis of the glycosyl-phosphatidylinositol membrane anchor of the merozoite surface proteins-1 and -2 of *Plasmodium falciparum*.

4. Cowman AF, Baldi DL, Healer J, et al. (2000) Functional analysis of proteins involved in *Plasmodium falciparum* merozoite invasion of red blood cells. *FEBS Lett 476:* 84-88.

5. [Dubbeld MA, Kocken CH, Thomas AW.](http://www.ncbi.nlm.nih.gov/entrez/query.fcgi?db=pubmed&cmd=Retrieve&dopt=AbstractPlus&list_uids=9574922&query_hl=20&itool=pubmed_docsum) (1998) Merozoite surface protein 2 of *Plasmodium reichenowi* is a unique mosaic of *Plasmodium falciparum* allelic forms and species-specific elements. *Mol* *Biochem Parasitol 92:* 187-92.

6. Smythe JA, Coppel RL, Day KP. et al. (1991) Structural diversity in the *Plasmodium falciparum* merozoite surface antigen 2. *Proc Natl Acad Sci USA 88:* 1751-1755.

7. [Fenton B, Clark JT, Khan CM, et al. (1991)](http://www.ncbi.nlm.nih.gov/entrez/query.fcgi?db=pubmed&cmd=Retrieve&dopt=AbstractPlus&list_uids=1990294&query_hl=21&itool=pubmed_docsum) Structural and antigenic polymorphism of the 35- to 48-kilodalton merozoite surface antigen (MSA-2) of the malaria parasite *Plasmodium falciparum.* *Mol Cell Biol. 11:* 963-71.

8. Al-Yaman F, Genton B, Anders RF, et al. (1994) Relationship between humoral response to *Plasmodium falciparum* merozoite surface antigen-2 and malaria morbidity in a highly endemic area of Papua New Guinea*. Am J Trop Med Hyg 51:* 593-602.

9. Taylor RR, Smith DB, Robinson VJ, et al. (1995) Human antibody response to *Plasmodium falciparum* merozoite surface protein 2 is serogroup specific and predominantly of the immunoglobulin G3 subclass. *Infect Immun 11:* 4382-8.

10. Lawrence N, Stowers A, Mann V, et al. (2000) Recombinant chimeric proteins generated from conserved regions of *Plasmodium falciparum* merozoite surface protein 2 generate antiparasite humoral responses in mice. *Parasite Immunol 22:* 211-221.

11. Taylor RR, Allen SJ, Greenwood BM, Riley EM. (1998) IgG3 antibodies to *Plasmodium falciparum* merozoite surface protein 2 (MSP2): increasing prevalence with age and association with clinical immunity to malaria. *Am J Trop Med Hyg 58:* 406-413.

12. Metzger WG, Okenu DM, Cavanagh DR, et al. (2003) Serum IgG3 to the *Plasmodium falciparum* merozoite surface protein 2 is strongly associated with a reduced prospective risk of malaria. *Parasite Immunol 25:* 307-312.

13. Polley SD, Conway DJ, Cavanagh DR, et al. (2006) High levels of serum antibodies to merozoite surface protein 2 of *Plasmodium falciparum* are associated with reduced risk of clinical malaria in coastal Kenya. *Vaccine 24:* 4233-4246.

14. [Eisen DP, Saul A, Fryauff DJ, Reeder JC, Coppel RL.](http://www.ncbi.nlm.nih.gov/entrez/query.fcgi?db=pubmed&cmd=Retrieve&dopt=AbstractPlus&list_uids=12363069&query_hl=17&itool=pubmed_docsum) (2002) Alterations in *Plasmodium falciparum* genotypes during sequential infections suggest the presence of strain specific immunity. *Am J Trop Med Hyg 67:* 8-16.

1. Epping RJ, [Goldstone SD](http://www.ncbi.nlm.nih.gov/entrez/query.fcgi?db=pubmed&cmd=Search&itool=pubmed_AbstractPlus&term="Goldstone+SD"%5BAuthor%5D), [Ingram LT](http://www.ncbi.nlm.nih.gov/entrez/query.fcgi?db=pubmed&cmd=Search&itool=pubmed_AbstractPlus&term="Ingram+LT"%5BAuthor%5D), et al. (1988) An epitope recognised by inhibitory monoclonal antibodies that react with a 51 kilodalton merozoite surface antigen in *Plasmodium falciparum*. *Mol. Biochem. Parasitol.* 1988, 28:1-10.

16. Clark JT, Donachie S, Anand R. et al. (1989) 46-53 kilodalton glycoprotein from the surface of *Plasmodium falciparum* merozoites *Mol. Biochem. Parasitol.* 1989, 32:15-24.

17. Miettinen-Baumann A, [Strych W](http://www.ncbi.nlm.nih.gov/entrez/query.fcgi?db=pubmed&cmd=Search&itool=pubmed_AbstractPlus&term="Strych+W"%5BAuthor%5D), [McBride J](http://www.ncbi.nlm.nih.gov/entrez/query.fcgi?db=pubmed&cmd=Search&itool=pubmed_AbstractPlus&term="McBride+J"%5BAuthor%5D), [Heidrich HG](http://www.ncbi.nlm.nih.gov/entrez/query.fcgi?db=pubmed&cmd=Search&itool=pubmed_AbstractPlus&term="Heidrich+HG"%5BAuthor%5D), et al. (1988) A 46,000 dalton *Plasmodium falciparum* merozoite surface glycoprotein not related to the 185,000-195,000 dalton schizont precursor molecule: isolation and characterization. *Parasitol. Res.* 1988, 74:317-323.

18. [Druilhe P, Bouharoun-Tayoun H.](http://www.ncbi.nlm.nih.gov/entrez/query.fcgi?db=pubmed&cmd=Retrieve&dopt=AbstractPlus&list_uids=12125151&query_hl=28&itool=pubmed_docsum) (2002) Antibody-dependent cellular inhibition assay. *Methods Mol Med 72:* 529-34.

19. Miles A, McClellan H, Rausch K, et al. (2005) Montanide ISA720 vaccines: quality control of emulsions, stability of formulated antigens, and comparative immunogenicity of vaccine formulations. *Vaccine 23:* 2530-39.

20. [Sturchler D, Berger R, Rudin C, et al. (1995)](http://www.ncbi.nlm.nih.gov/entrez/query.fcgi?db=pubmed&cmd=Retrieve&dopt=AbstractPlus&list_uids=7485698&query_hl=8&itool=pubmed_docsum) Safety, immunogenicity, and pilot efficacy of Plasmodium falciparum sporozoite and asexual blood-stage combination vaccine in Swiss adults. *Am J Trop Med Hyg 53:* 423-31.

21. [Saul A, Lawrence G, Smillie A, et al. (1999)](http://www.ncbi.nlm.nih.gov/entrez/query.fcgi?db=pubmed&cmd=Retrieve&dopt=AbstractPlus&list_uids=10462251&query_hl=8&itool=pubmed_docsum) Human phase I vaccine trials of 3 recombinant asexual stage malaria antigens with Montanide ISA720 adjuvant. *Vaccine 17:* 3145-59.

22. [Lawrence G, Cheng QQ, Reed C, et al. (2000)](http://www.ncbi.nlm.nih.gov/entrez/query.fcgi?db=pubmed&cmd=Retrieve&dopt=AbstractPlus&list_uids=10699342&query_hl=8&itool=pubmed_docsum) Effect of vaccination with 3 recombinant asexual-stage malaria antigens on initial growth rates of *Plasmodium falciparum* in non-immune volunteers. *Vaccine 18:* 1925-31.

23. Cheng Q, Lawrence G, Reed C, et al. (1997) Measurement of *Plasmodium falciparum* growth rates in vivo: a test of malaria vaccines. *Am J* *Trop Med Hyg 57:* 495-500.

24. [Genton B, Al-Yaman F, Anders R, et al. (2000)](http://www.ncbi.nlm.nih.gov/entrez/query.fcgi?db=pubmed&cmd=Retrieve&dopt=AbstractPlus&list_uids=10775784&query_hl=8&itool=pubmed_docsum) Safety and immunogenicity of a three-component blood-stage malaria vaccine in adults living in an endemic area of Papua New Guinea*. Vaccine 18:* 2504-11.

25. [Genton B, Betuela I, Felger I, Al-Yaman F, et al. (2002)](http://www.ncbi.nlm.nih.gov/entrez/query.fcgi?db=pubmed&cmd=Retrieve&dopt=AbstractPlus&list_uids=11920300&query_hl=8&itool=pubmed_docsum) A recombinant blood-stage malaria vaccine reduces Plasmodium falciparum density and exerts selective pressure on parasite populations in a phase 1-2b trial in Papua New Guinea. *J Infect Dis 185:* 820-7.

26. [Genton B](http://www.ncbi.nlm.nih.gov/entrez/query.fcgi?db=pubmed&cmd=Search&itool=pubmed_AbstractPlus&term="Genton+B"%5BAuthor%5D), [Al-Yaman F](http://www.ncbi.nlm.nih.gov/entrez/query.fcgi?db=pubmed&cmd=Search&itool=pubmed_AbstractPlus&term="Al-Yaman+F"%5BAuthor%5D), [Betuela I](http://www.ncbi.nlm.nih.gov/entrez/query.fcgi?db=pubmed&cmd=Search&itool=pubmed_AbstractPlus&term="Betuela+I"%5BAuthor%5D), et al. (2003) Safety and immunogenicity of a three-component blood-stage malaria vaccine (MSP1, MSP2, RESA) against *Plasmodium falciparum* in Papua New Guinean children. *Vaccine 22:* 30-41.

27. Lopez JA, Weilenman C, Audran R, et al. (2001) A synthetic malaria vaccine elicits a potent CD8+ and CD4+ T lymphocyte immune response in humans. Implication for vaccination strategies. *Eur J Immunol 31:* 1989-98.

28. Saul A, Lawrence G, Allworth A, et al. (2005) A human phase 1 vaccine clinical trial of the *Plasmodium falciparum* malaria vaccine candidate apical membrane antigen 1 in Montanide ISA720 adjuvant. *Vaccine 23:* 3076-83.

29. [Hermsen CC](http://www.ncbi.nlm.nih.gov/entrez/query.fcgi?db=pubmed&cmd=Search&itool=pubmed_AbstractPlus&term="Hermsen+CC"%5BAuthor%5D), [Verhage DF](http://www.ncbi.nlm.nih.gov/entrez/query.fcgi?db=pubmed&cmd=Search&itool=pubmed_AbstractPlus&term="Verhage+DF"%5BAuthor%5D), [Telgt DS](http://www.ncbi.nlm.nih.gov/entrez/query.fcgi?db=pubmed&cmd=Search&itool=pubmed_AbstractPlus&term="Telgt+DS"%5BAuthor%5D), et al. (2007) Glutamate-rich protein (GLURP) induces antibodies that inhibit in vitro growth of *Plasmodium falciparum* in a phase 1 malaria vaccine trial. *Vaccine 25:* 2930-40.

30. [Audran R](http://www.ncbi.nlm.nih.gov/entrez/query.fcgi?db=pubmed&cmd=Search&itool=pubmed_AbstractPlus&term="Audran+R"%5BAuthor%5D), [Cachat M](http://www.ncbi.nlm.nih.gov/entrez/query.fcgi?db=pubmed&cmd=Search&itool=pubmed_AbstractPlus&term="Cachat+M"%5BAuthor%5D), [Lurati F](http://www.ncbi.nlm.nih.gov/entrez/query.fcgi?db=pubmed&cmd=Search&itool=pubmed_AbstractPlus&term="Lurati+F"%5BAuthor%5D), et al. (2005) Phase I malaria vaccine trial with a long synthetic peptide derived from the merozoite surface protein 3 antigen. *Infect Immun 73:* 8017-26.

31. Oliveira GA, [Wetzel K](http://www.ncbi.nlm.nih.gov/entrez/query.fcgi?db=pubmed&cmd=Search&itool=pubmed_AbstractPlus&term="Wetzel+K"%5BAuthor%5D), [Calvo-Calle JM](http://www.ncbi.nlm.nih.gov/entrez/query.fcgi?db=pubmed&cmd=Search&itool=pubmed_AbstractPlus&term="Calvo-Calle+JM"%5BAuthor%5D), et al. (2005) Safety and enhanced immunogenicity of a hepatitis B core particle *Plasmodium falciparum* malaria vaccine formulated in adjuvant Montanide ISA 720 in a phase I trial. *Infect Immun 73:* 3587-97

32. [Walther M](http://www.ncbi.nlm.nih.gov/entrez/query.fcgi?db=pubmed&cmd=Search&itool=pubmed_AbstractPlus&term="Walther+M"%5BAuthor%5D), [Dunachie S](http://www.ncbi.nlm.nih.gov/entrez/query.fcgi?db=pubmed&cmd=Search&itool=pubmed_AbstractPlus&term="Dunachie+S"%5BAuthor%5D), [Keating S](http://www.ncbi.nlm.nih.gov/entrez/query.fcgi?db=pubmed&cmd=Search&itool=pubmed_AbstractPlus&term="Keating+S"%5BAuthor%5D), et al. (2005) Safety, immunogenicity and efficacy of a pre-erythrocytic malaria candidate vaccine, ICC-1132 formulated in Seppic ISA 720. *Vaccine 23:* 857-64.

33. Toledo H, Baly A, Castro O, et al. (2001) A phase I clinical trial of a multi-epitope polypeptide TAB9 combined with Montanide ISA720 adjuvant in non-HIV-1 infected human volunteers. *Vaccine 19:* 4328-36.

34. [Engers H, Kieny MP, Malhotra P, Pink JR.](http://www.ncbi.nlm.nih.gov/sites/entrez?Db=pubmed&Cmd=ShowDetailView&TermToSearch=14518430&ordinalpos=1&itool=EntrezSystem2.PEntrez.Pubmed.Pubmed_ResultsPanel.Pubmed_RVDocSum) (2003) Third meeting on Novel Adjuvants Currently in or Close to Clinical Testing World Health Organization--Organisation Mondiale de la Sante, Fondation Merieux, Annecy, France, 7-9 January 2002. *Vaccine 21:*3503-24.

35. Cody CL, Baraff LJ, Cherry JD, Marcy SM & Manclark CR. (1981) Nature and rates of adverse reactions associated with DTP and DT immunizations in infants and children. *Pediatrics 68:*650-660.

36. Persson KEM, Lee CT, Marsh K and Beeson JG. (2006) The development and optimization of high throughput methods to measure Plasmodium falciparum growth inhibitory antibodies. *J.* *Clin. Microbiol. 44:*1665-73)

37. Holm S. A simple sequentially rejective multiple test procedure. (1979) *Scandinavian Journal of Statistics, 6:*65–70.

38. Patterson H and Thompson R. (1971) Recovery of inter-block information when block sizes are unequal. *Biometrika, 58:*545–54.

Appendices

Schedule of Assessments

Assessment of Adverse Event Severity

Severity Tables for Grading Laboratory Adverse Events

Tables for Grading Adverse Events

Appendix 1 - MSP2 Schedule of Events

| **Procedures** | **WK** |  | **0** |  |  | **1** | **2** |  | **4** | **8** | **12** |  |  | **13** | **14** | **15** | **16** | **20** |
| --- | --- | --- | --- | --- | --- | --- | --- | --- | --- | --- | --- | --- | --- | --- | --- | --- | --- | --- |
|  | **Day** | **Screen** | **0** | **1** | **3** | **7** | **14** | **21** | **28** | **56** | **84** | **85** | **87** | **91** | **98** | **105** | **112** | **140** |
| Obtain Informed Consent |  | X^1^ | X^2^ |  |  |  |  |  |  |  |  |  |  |  |  |  |  |  |
| Complete History and Physical |  | X |  |  |  |  |  |  |  |  |  |  |  |  |  |  |  |  |
| Clinical Evaluation |  |  | X |  | X | X | X |  | X | X | X |  | X | X | X |  | X | X |
| Distribute diary card |  |  | X |  |  | X | X |  |  |  | X |  |  | X | X |  |  |  |
| Phone call |  |  |  | X |  |  |  | X |  |  |  | X |  |  |  | X |  |  |
| Collect diary card |  |  |  |  |  | X | X |  | X |  |  |  |  | X | X |  | X |  |
| FBC & Diff, Biochem (9 mL) (14 ml) 5 |  | X | X5 |  | X |  | X5 |  |  | X | X5 |  | X |  | X5 |  |  | X |
| Urinalysis |  | X |  |  |  |  |  |  |  |  |  |  |  |  |  |  |  |  |
| Pregnancy test^3^ (females) |  | X* | X |  |  |  |  |  |  |  | X |  |  |  |  |  |  |  |
| HIV HCV, HBsAg  (10 mL) |  | X |  |  |  |  |  |  |  |  |  |  |  |  |  |  |  |  |
| **VACCINATION** |  |  | **X** |  |  |  |  |  |  |  | **X** |  |  |  |  |  |  |  |
| Anti-MSP2 antibody ELISA  (10 mL) |  |  | X |  |  |  |  |  | X |  | X |  |  |  |  |  | X |  |
| Cell Mediated Immunity  (45 ml=40 mL [PBMC] + 5 ml [serum]) |  | X |  |  |  |  |  |  | X |  |  |  |  |  |  |  | X |  |
| Growth Inhibition Assay (GIA ) (from CMI plasma 2 mL for NIH assay) |  | X |  |  |  |  |  |  |  |  |  |  |  |  |  |  | X |  |
| Immunoflorescence Assay (IFA) with Ab (supernatent plasma off CMI whole blood) |  | X |  |  |  |  |  |  |  |  |  |  |  |  |  |  | X |  |
| Blood Volume (mL) |  | 64 | 19 |  | 9 |  | 14 |  | 55 | 9 | 19 |  | 9 |  | 14 | 9 | 55 | 9 |
| Cumulative Blood Volume (mL) |  | 64 | 83 |  | 92 |  | 106 |  | 160 | 169 | 189 |  | 193 |  | 212 | 221 | 276 | 285 |

**^1^ *Screening Consent,* ^2^*Particpation Consent,* ^3^ *Screening pregnancy (female only) = Serum Β-HCG (+10 mL blood) , ^4^Biochemistry to include: creatinine, AST and ALT,^5^ with CPK***

Appendix 1 - MSP2 Schedule of Events

| **Procedures** | **WK** | **24** |  |  | **25** | **26** |  | **28** | **48** |
| --- | --- | --- | --- | --- | --- | --- | --- | --- | --- |
|  | **Day** | **168** | **169** | **171** | **175** | **182** | **189** | **196** | **336** |
| Clinical Evaluation |  | X |  | X | X | X |  | X | X |
| Distribute Diary Card |  | X |  |  | X | X |  |  |  |
| Phone Call |  |  | X |  |  |  | X |  |  |
| Collect Diary Card |  |  |  |  | X | X |  | X |  |
| FBC & Diff, Biochem (9 mL) (14)^5^ |  | X5 |  | X |  | X5 |  |  |  |
| Urine pregnancy test (females) |  | X |  |  |  |  |  |  |  |
| **VACCINATION** |  | **X** |  |  |  |  |  |  |  |
| Anti-MSP2 antibody ELISA (10 mL) |  | X |  |  |  |  |  | X | X |
| Cell Mediated Immunity (45 ml=40 mL [PBMC] + 5 ml [serum]) |  |  |  |  |  |  |  | X | X |
| Growth Inhibition Assay (GIA ) (from CMI plasma 2 mL for NIH assay) |  |  |  |  |  |  |  | X | X |
| Immunoflorescence Assay (IFA) with Ab (supernatent plasma off CMI whole blood) |  |  |  |  |  |  |  | X | X |
| Blood Volume (mL) |  | 19 |  | 9 |  | 14 |  | 55 | 55 |
| Cumulative Blood Volume (mL) | 285 | 304 |  | 313 |  | 327 |  | 382 | 442 |

^1^ *Screening Consent,* ^2^*Particpation Consent,* ^3^ *Screening pregnancy (female only) = Serum Β-HCG (+10 mL blood) , ^4^Biochemistry to include: creatinine, AST and ALT,^5^ with CPK*

Appendix 2 - Assessment of Adverse Event Severity

| **Adverse Event** | **Grade** | **Intensity** |
| --- | --- | --- |
| Pain at injection site | 0 | Absent |
|  | 1 | Pain that is easily tolerated |
|  | 2 | Pain that interferes with daily activity |
|  | 3 | Pain that prevents daily activity |
| Erythema at injection site | 0 | 0 mm |
|  | 1 | >0 - <20 mm |
|  | 2 | >20 - <50 mm |
|  | 3 | >50 mm |
| Swelling at injection site | 0 | 0 mm |
|  | 1 | >0 - <20 mm |
|  | 2 | >20 - <50 mm |
|  | 3 | >50 mm |
| Induration at injection site | 0 | 0 mm |
|  | 1 | >0 - <20 mm |
|  | 2 | >20 - <50 mm |
|  | 3 | >50 mm |
| Fever (oral) | 0 | <37.5°C |
|  | 1 | 37.6°C - 38.0°C |
|  | 2 | >38.0°C – 39.0°C |
|  | 3 | >39.0°C |
| Headache | 0 | None |
|  | 1 | Headache that is easily tolerated |
|  | 2 | Headache that interferes with daily activity |
|  | 3 | Headache that prevents daily activity |
| Nausea | 0 | None |
|  | 1 | Nausea that is easily tolerated |
|  | 2 | Nausea that interferes with daily activity |
|  | 3 | Nausea that prevents daily activity |
| Malaise | 0 | None |
|  | 1 | Malaise that is easily tolerated |
|  | 2 | Malaise that interferes with daily activity |
|  | 3 | Malaise that prevents daily activity |
| Myalgia | 0 | None |
|  | 1 | Myalgia that is easily tolerated |
|  | 2 | Myalgia that interferes with daily activity |
|  | 3 | Myalgia that prevents daily activity |
| Arthralgia | 0 | None |
|  | 1 | Joint pain that is easily tolerated |
|  | 2 | Joint pain that interferes with daily activity |
|  | 3 | Joint pain that prevents daily activity |
| Urticaria | 0 | None |
|  | 1 | Requiring no medications |
|  | 2 | Requiring PO or topical treatment or IV medication or steroids for <24 hours |
|  | 3 | Requiring IV medication or steroids for >24 hours |

Appendix 3 – Severity Tables for Grading Laboratory Adverse Events

These tables are to be used to assess laboratory adverse events for those tests to be performed as part of the MSP2-C1/ISA720 malaria vaccine clinical trial protocol.

**ABBREVIATIONS:** ULN = Upper Limit of Normal and LLN = Lower Limit of Normal of the testing laboratory

| **Laboratory Test** | **Grade 1** | **Grade 2** | **Grade 3** | **Grade 4  (Life threatening)** |
| --- | --- | --- | --- | --- |
| Hb (female) – decrease from LLN | 1.0 - <1.5 | ≥1.5 & <2.0 | ≥2.0 | Requires transfusion |
| Hb (male) – decrease from LLN | ≥1.5 & <2.0 | ≥2.0 & <2.5 | ≥2.5 | Requires transfusion |
| Absolute neutrophil count (ANC, x10^9^cells/L)) | 1.00-1.49 | 0.50-0.99 | <0.50 | <0.50 with fever |
| Leukopenia (WBC, x10^9^cells/L) | <3.50 - ≥2.50 | <2.50 - ≥1.50 | <1.50 | <1.50 with fever |
| Platelets (x10^9^cells/L)) | 125 – 135 | 100 – 124 | 20-99 | <20 |
| ALT  and  AST | 1.25 – 2.5 x ULN | >2.6 – 5.0 x ULN | >5.0 ULN | >10 x ULN and requires hospitalization |
| Creatinine | 1.1 – 1.5 x ULN | >1.6 – 3.0 x ULN | >3.0 ULN | >5.0 x ULN and requires dialysis |
| Creatine Phosphokinase | 1.25-1.5 x ULN | 1.6-3.0 x ULN | 3.1-10 x ULN | >10 x ULN |
| Urine protein | 2+ or 0.5-1 gm loss/day | 3+ or 1-2 gm loss/day | 4+ or >2 gm loss/day | NA |
| Hematuria | 2+ confirmed by 5-10 rbc/hpf | 3+ confirmed by >10 rbc/hpf | gross, with or without clots, OR red blood cell casts | NA |

Appendix 4– Tables for Grading Adverse Events

These tables are to be used to grade unexpected adverse events not described in Appendices 2 and 3.

| **Vital Signs*** | **Grade 1**  **(Mild)** | **Grade 2**  **(Moderate)** | **Grade 3 (Severe)** | **Grade 4**  **(Life threatening)** |
| --- | --- | --- | --- | --- |
| Tachycardia –  beats per minute | 110-120 | 121-140 | >140 | >160 with symptoms** |
| Bradycardia –  beats per minute | 50-54 | 45-49 | <45 | <35 with loss of consciousness |
| Hypertension (systolic) – mm Hg (with repeat testing at same visit) | 141-150 | 151-170 | >170 | >200 with symptoms** |
| Hypertension (diastolic) –  mm Hg (with repeat testing at same visit) | 91-100 | 101-110 | >110 | >120 with symptoms** |
| Hypotension (systolic) –  mm Hg (with repeat testing at same visit) | 85-89 (and symptomatic) | 80-84 (and symptomatic) | <80 | < 70 with loss of consciousness |
| * Participant should be at rest for measurement of vital signs  ** Severe headache, chest pain, altered consciousness, or other signs of acute organ damage | | | | |

| **Systemic** | **Grade 1 (Mild)** | **Grade 2  (Moderate)** | **Grade 3 (Severe)** | **Grade 4  (Life threatening)** |
| --- | --- | --- | --- | --- |
| Anorexia | Loss of appetite without decreased oral intake lasting greater than 48 hours | Loss of appetite associated with decreased oral intake without significant weight loss | Loss of appetite associated with significant weight loss | NA |
| Vomiting | 1-2 episodes/24 hours | > 2 episodes/24 hours | Prevents daily activity, requires outpatient IV hydration | Requires hospitalization |
| Diarrhea | 2-3 loose stools/24 hours | 4-5 loose stools/24 hours | >6 loose stools or requires outpatient IV hydration | Requires hospitalization |
| Constipation | NA | Persistent constipation requiring regular use of dietary modifications, laxatives, or enemas | Obstipation with manual evacuation indicated | NA |
| Fatigue | No interference w/activity | Some interference w/activity | Significant, prevents daily activity | NA |
| Arthritis | Mild pain with inflammation, erythema or joint swelling – but not interfering with function | Moderate pain with inflammation, erythema or joint swelling – interfering with function, but not with activities of daily living | Severe pain with inflammation, erythema or joint swelling –and interfering with activities of daily living | NA |
| Mucocutaneous Reaction/Rash | Erythema; pruritis or localized macular rash | Diffuse, maculo-papular rash, dry desquamation | Vesiculation or moist desquamation or ulceration | Requires hospitalisation |
| Vasovagal episode (associated with a procedure of any kind) | Present without loss of consciousness | Present with transient loss of consciousness | NA | NA |
| Vertigo | Causes no or minimal interference with usual daily activities | Causes greater than minimal interference with usual daily activities | Inability to perform daily activities | NA |
| Cough | transient- no treatment | persistent cough; treatment responsive | Paroxysmal cough; uncontrolled with treatment | Requires hospitalization |
| Bronchospasm, Acute | transient; no treatment;  70% - 80% FEV_1_  of peak flow | requires treatment; normalizes with bronchodilator;  FEV_1_ 50% - 70%  (of peak flow) | no normalization with bronchodilator;  FEV_1_ 25% - 50%  of peak flow; or retractions present | Requires hospitalisation |
| Dyspnoea | Dyspnoea on exertion | Dyspnoea with normal activity | Dyspnoea at rest | Requires hospitalisation |
