# Supplementary figures and images for: A Phase 1 Trial of MSP2-C1, a Blood-Stage Malaria Vaccine Containing 2 Isoforms of MSP2 Formulated with Montanide® ISA 720
Source: PLoS One. 2011 Sep 19;6(9):e24413. doi: 10.1371/journal.pone.0024413 (PMC3176224; doi:10.1371/journal.pone.0024413)

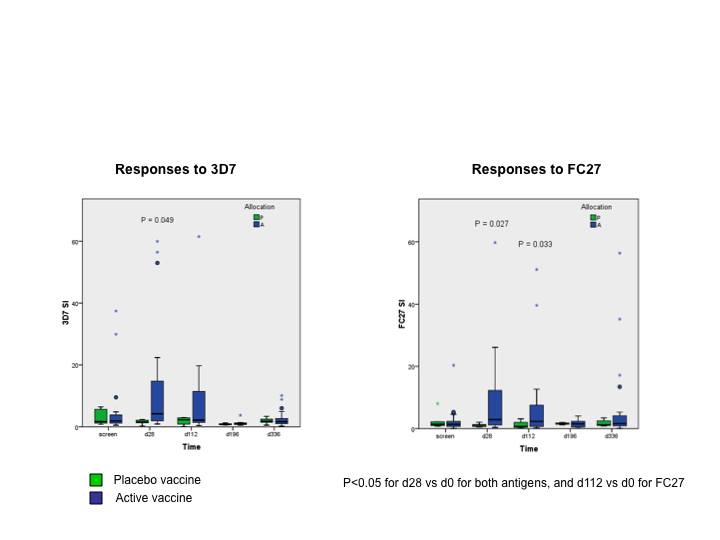

Supplement: Figure S1 — Lymphoproliferative responses to recombinant MSP2 proteins. Peripheral blood mononuclear cells were isolated from blood samples and tested in lymphoproliferation assays for responses to recombinant MSP2 of the 3D7 and FC27 alleles. Results are expressed as the stimulation index (SI), presented as box-and whisker plots showing the median (horizontal line), inter-quartile range (box at each time point for subjects receiving the active versus placebo vaccine. ‘Screen’ indicated the day 0 sample. The numbers for each comparison for are as follows: screen v d28, n = 19; screen v d112, n = 19. (TIF) [file pone.0024413.s006.tif]
