# Supplementary material for: A Phase 1 Trial of MSP2-C1, a Blood-Stage Malaria Vaccine Containing 2 Isoforms of MSP2 Formulated with Montanide® ISA 720
Source: PLoS One. 2011 Sep 19;6(9):e24413. doi: 10.1371/journal.pone.0024413 (PMC3176224; doi:10.1371/journal.pone.0024413)
Supplement: Table S1 — Two day 0 samples (marked with an asterisk) had substantial ADCI activity (70% and 76% for 3D7), and two day 112 samples (marked with an asterisk) had relatively low activity (27% and 42% for 3D7). In exploratory assays these four samples were retested in a blinded manner in ADCI assays, together with their matched day 0 or day 112 samples. Results show the adjusted ADCI values from the original assay and the repeat assay. Shown here is one example of two such re-test experiments, both of which yielded consistent results. All assays were performed with the P. falciparum 3D7 line using purified IgG. (DOC) [file pone.0024413.s007.doc]

**Table S1**. Retesting of selected samples in ADCI assays

| Subject | Day 0 | | Day 112 | |
| --- | --- | --- | --- | --- |
|  | First assay | Retest | First assay | Retest |
| 10 | 34% | 27% | 27%* | 93% |
| 17 | 26% | 36% | 42%* | 77% |
| 20 | 70%* | 24% | 90% | 76% |
| 28 | 76%* | 32% | 81% | 74% |
